# Comparative transcriptomics reveals hidden issues in the plant response to arthropod herbivores

**DOI:** 10.1111/jipb.13026

**Published:** 2021-02-12

**Authors:** M. Estrella Santamaria, Alejandro Garcia, Ana Arnaiz, Irene Rosa‐Diaz, Gara Romero‐Hernandez, Isabel Diaz, Manuel Martinez

**Affiliations:** ^1^ Centro de Biotecnología y Genómica de Plantas, Instituto Nacional de Investigación y Tecnología Agraria y Alimentaria Universidad Politécnica de Madrid Madrid Spain; ^2^ Departamento de Biotecnología‐Biología Vegetal, Escuela Técnica Superior de Ingeniería Agronómica, Alimentaria y de Biosistemas Universidad Politécnica de Madrid Madrid Spain

**Keywords:** *Arabidopsis thaliana*, arthropod herbivore, comparative transcriptomics, plant defense, *Tetranychus urticae*

## Abstract

Plants experience different abiotic/biotic stresses, which trigger their molecular machinery to cope with them. Besides general mechanisms prompted by many stresses, specific mechanisms have been introduced to optimize the response to individual threats. However, these key mechanisms are difficult to identify. Here, we introduce an in‐depth species‐specific transcriptomic analysis and conduct an extensive meta‐analysis of the responses to related species to gain more knowledge about plant responses. The spider mite *Tetranychus urticae* was used as the individual species, several arthropod herbivores as the related species for meta‐analysis, and *Arabidopsis thaliana* plants as the common host. The analysis of the transcriptomic data showed typical common responses to herbivory, such as jasmonate signaling or glucosinolate biosynthesis. Also, a specific set of genes likely involved in the particularities of the *Arabidopsis*‐spider mite interaction was discovered. The new findings have determined a prominent role in this interaction of the jasmonate‐induced pathways leading to the biosynthesis of anthocyanins and tocopherols. Therefore, tandem individual/general transcriptomic profiling has been revealed as an effective method to identify novel relevant processes and specificities in the plant response to environmental stresses.

## Graphical abstract

An in‐depth, species‐specific transcriptomic analysis and meta‐analysis of the responses to related species improves our understanding of plant responses to arthropod herbivores. This tandem individual/general transcriptomic profiling provides an effective method to identify novel relevant processes and specificities.

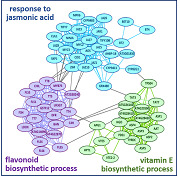

## INTRODUCTION

Plants are organisms subjected to direct and constant interaction with a broad range of stresses present in the environment. Exposure of plants to these stresses induces a disruption in the plant metabolism which leads to a reduction in their fitness and productivity (Rejeb et al., 2014). To cope adequately with these stresses, plants have developed specific mechanisms of resistance which allow them to detect precise environmental changes and respond to undesirable stress conditions. Among biotic cues, arthropod herbivores pose a widespread threat to plants that in the current context of climate change is becoming even more extreme. Climatic warming helps spread pest distribution, accelerate their life cycles, and increase the range of host species for many herbivores (DeLucia et al., 2012). Understanding the mechanisms of how plants are able to detect and recognize a stress, and act against it, is of prime importance in providing opportunities for the establishment of alternative strategies of control.

In this scene, the proper identification and characterization of the genes involved in plant response are crucial. Nowadays, the most popular and direct approach to decipher transcriptomic data is the gene differential expression analysis. Detection of differentially expressed genes (DEGs) represents a powerful way to perform a screening of plant defense‐related genes (Schenk et al., 2000; Korth, 2003). The availability of these data is fundamental in accelerating the study of gene functions in plant defense responses. For this reason, gene expression databases have been generated to provide public access. Gene Expression Omnibus and ArrayExpress are two of the most popular repositories of high throughput gene expression data (Edgar et al., 2002; Parkinson et al., 2007). Nevertheless, the use of this information for broad gene expression comparisons remains not a trivial task. Inherent to their underlying technology, microarray data are not as comprehensive as RNA‐seq data and not all genes are represented on microarrays. The processed data may not be directly comparable since expression abundance values are provided in different data formats. Furthermore, annotation of the genes can differ across different experiments, making automatic parsing processes not straightforward. Despite that, several approaches to identify functional modules or genes have pointed out the value of stored data for comparative transcriptomics analysis (Ruprecht et al., 2017; Vercruysse et al., 2020). In this scenario, the development of specialized transcriptomics databases where standards are implemented is highly required (Stoeckert et al., 2002; Rung and Brazma, 2013).

Some attempts have been done to create transcriptomics databases for the analysis of the plant immune response, mostly focused on pathogen experiments. For instance, ExPath (Chien et al., 2015) and PathoPlant (Bülow et al., 2004) are two transcriptomics databases for analyzing co‐regulated genes in plant defense response. Other examples are the plant stress RNA‐seq database Nexus, a stress‐specific transcriptome database in plant cells (Li et al., 2018) and PlaD, a database where 2,444 public pathogenesis‐related gene expression samples from *Arabidopsis*, maize, rice and wheat have been analyzed in a similar way to perform comparisons across the different samples (Qi et al., 2018). Regarding arthropod experiments, even less information has been reported, which essentially comes from individual analyses of the transcriptomic response of a plant to a herbivore. Additional useful databases related to the process of recognition of biotic agents have been developed, like the Plant Resistance Gene database 3.0 (PRGdb) (Osuna‐Cruz et al., 2018). Nonetheless, despite the considerable amount of information obtained in various studies related to changes in plant gene expression, it is still not deeply understood how plants recognize a particular stress and respond in a rapid way. Deep transcriptomic analysis to an individual species together with a broad meta‐analysis of the transcriptomic responses to related species could help go further in this direction.

The interaction between the two‐spotted spider mite *Tetranychus urticae* and the model plant *Arabidopsis thaliana* has become a good system to adequately explore the response of the plant to a herbivore attack (Santamaria et al., 2020). Thus, starting from a comprehensive analysis of the transcriptomic *Arabidopsis* response to the spider mite, this work is focused on the capacity of comparative transcriptomics to discover additional key points in this response. Taking together our experimental results and the information generated by the meta‐analysis, unknown processes and particularities of the plant response to the spider mite were revealed.

## RESULTS

### DEGs upon different times of spider mite infestation

The development of the early response of *Arabidopsis* plants to *T. urticae* infestation was assessed by the transcriptomic analysis at 30 min, 1, 3, and 24 h of infestation in leaves of the partially resistant Col‐0 accession. Principal components analysis (PCA) showed good separation of the samples coming from the three biological replicates in time‐related groups using the first two components (Figure S1A). A first insight on the differential response along the time of infestation came from the variations in the percentage of reads per chromosome. This percentage decreased in chromosomes 1, 4, and 5, increasing in chromosomes 2 and 3 as well as in the mitochondrial and plastid chromosomes (Figure S1B). These changes were not associated with variations in the amount of paired reads or the number of detected genes with at least one read mapped (Figure S1C, D).

**Figure 1:**
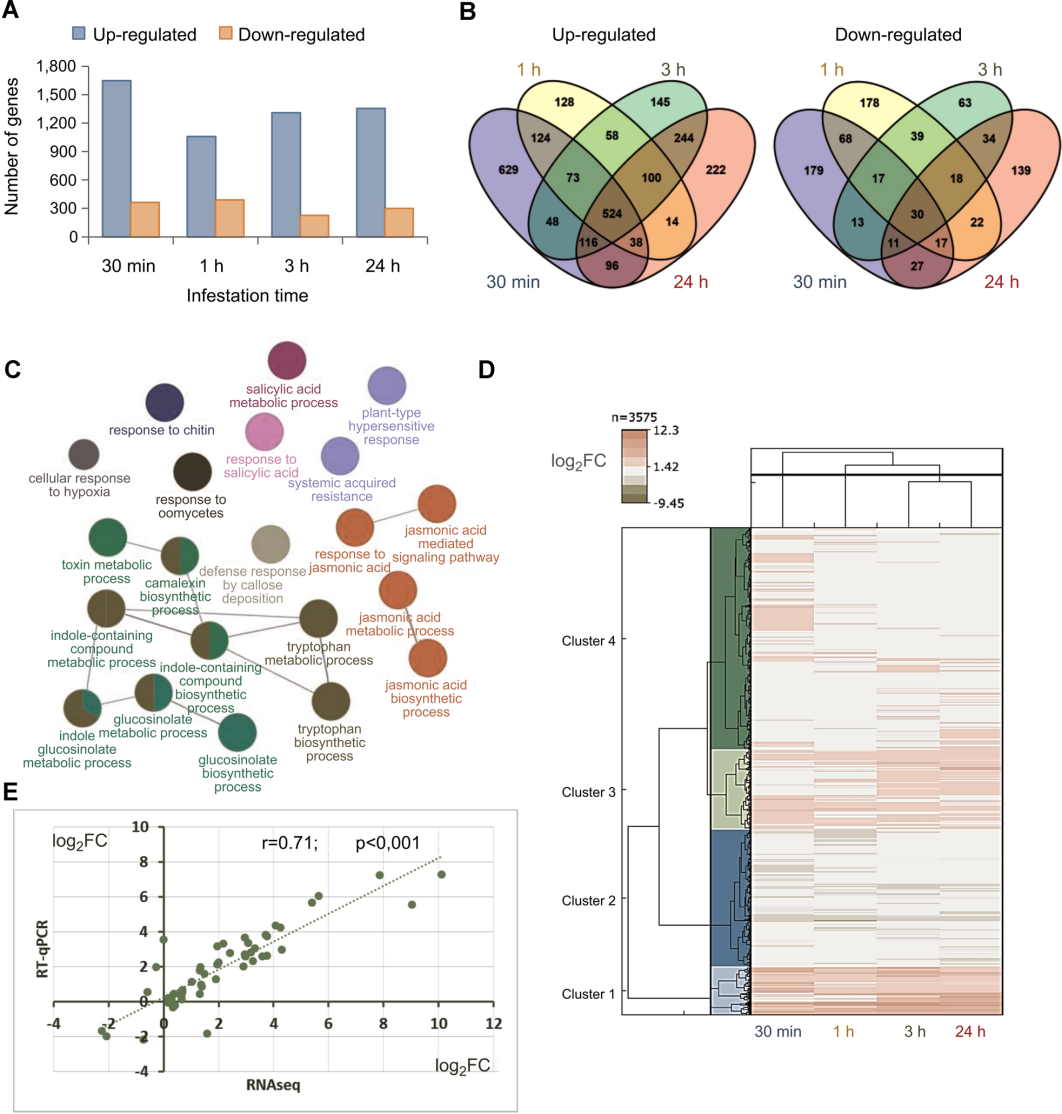
**Transcriptional analysis of *Arabidopsis* plants infested with *Tetranychus urticae* during 30 min, 1, 3, and 24 h** (**A**) Bar diagrams showing the number of up‐ or down‐regulated genes at each infestation time point. (**B**) Venn diagrams showing the number of specific and shared up‐ or down‐regulated genes among infestation time points. (**C**) Network of the most enriched biological processes using all detected differentially expressed genes (DEGs) upon mite infestation. Colors correspond to groups of connected related terms. (**D**) Heatmap showing hierarchical clustering analysis of the log_2_FC values exhibited by the 3,575 DEGs found in mite‐infested plants compared to control plants. (**E**) Dispersion graphic showing the correlation between the log_2_FC values from RNA‐seq and quantitative real‐time polymerase chain reaction assays for 15 selected genes. Spearman rank correlation coefficient (r) is showed.

Differentially expressed genes were obtained for each time point (Data S1). The number of up‐regulated genes was higher than the number of down‐regulated genes for all the time points (Figure 1A). An analysis of the overlaps of DEGs along time showed a remarkably high number of genes only up‐regulated at 30 min, 629; a substantial set of genes up‐regulated in all time points, 524; and a minor number of genes only up‐regulated at 24 h, 222. By contrast, down‐regulated genes did not present any particular pattern (Figure 1B; Data S2). All detected DEGs were used to identify the enriched biological processes in the plant triggered during mite infestation. As expected, ontology terms related to defensive processes were predominantly found (Figure 1C). These terms included those associated with the jasmonic acid signaling and the indole glucosinolate metabolism. The relationship between expression of DEGs and time of infestation is highlighted in a heatmap (Figure 1D). Whereas many genes up‐regulated in the four time points are grouped in cluster 1, a large group of genes specifically up‐regulated at 30 min is present in cluster 4. A close correlation between RNA‐seq and quantitative real‐time polymerase chain reaction (RT‐qPCR) results was observed for the 15 genes tested (Figures 1E, S2).

### Temporal regulation of gene modules upon spider mite infestation

**Figure 2:**
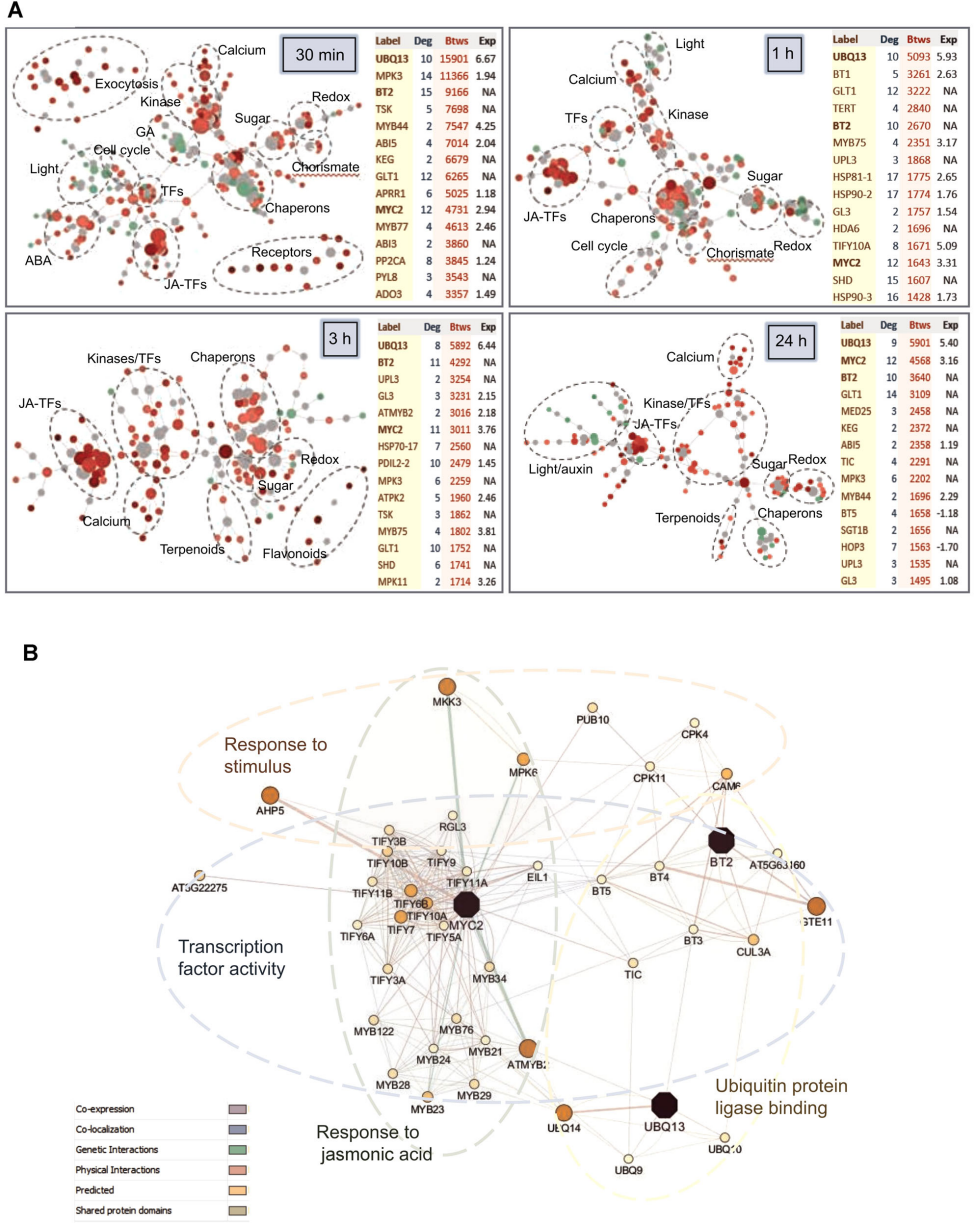
**Analysis of the minimum connected molecular networks** (**A**) Minimum connected molecular networks based on the differentially expressed genes (DEGs) in each time point and the protein‐protein interactions from STRING database. Red points, up‐regulated genes; green points, down‐regulated genes; gray points, non‐seed genes. Ellipses mark groups of genes functionally related. Tables show the 15 genes with the highest values of betweenness (Btws). The main genes detected in all infestation times are boldly typed. Deg, degree. Exp, expression fold change. NA, not altered. (**B**) Minimum gene network connecting the three main genes (purple). Size and colors of nodes are based on the GeneMANIA scores. Width of edges depends on the normalized maximum weight value.

The specificity in the up‐regulation of many genes at different time points suggested the possibility of a concomitant dissimilarity in the activation of gene modules. To characterize the temporal similarities/differences in the regulation of specific gene modules, gene networks were performed using the NetworkAnalyst software (Figure 2A). The obtained networks comprise a mix of interactions between induced and repressed genes, as well as additional interactions with non‐regulated genes necessary to connect regulated nodes that share a common putative interactor (Data S3). From these networks, a time‐associated pattern arose. Modules of genes related to signal perception and transduction, exocytosis enhancement, and jasmonate defense were rapidly altered, as well as modules of genes related to the control of the cell cycle and light and hormonal responses associated with growth and development. From 3 h of infestation, modules related to the production of secondary metabolites, such as terpenoids and flavonoids, were identified. Upon 24 h of infestation, alterations in genes involved in development were detected again. Further, groups of genes related to sugar transport, redox modulation, and protein folding were identified at the four times of infestation as a part of the connected networks. An analysis of the enriched biological processes for the DEGs at each time point largely agrees with the identified modules (Table S1).

### Generation of a core gene framework involved in plant defense against *T. urticae*

In an attempt to simplify the complex response of the plant to the mite infestation, a new network was constructed based on the centrality features of the networks previously created. The 15 genes with the top betweenness values were selected from each time point (Figure 2A). Betweenness centrality is based on the shortest paths connecting network genes and a high value for a gene indicates that this node is a hub necessary to connect various branches of the network. As shown in Figure 2A, betweenness and degree, the number of connections of each node, exhibited a low correlation. Further, many of the genes with the top betweenness values were not differentially expressed upon mite infestation but were required to construct the connected network. From the analysis of the gene lists, three genes were identified with elevated values of betweenness at the four time points. These genes were *UBQ13* and *MYC2*, which were always up‐regulated, and *BT2*, a gene that did not alter its expression in response to the spider mite. Using these three genes as seed, a core gene framework was constructed using the GeneMANIA database (Figure 2B). This network represents an initial identification of the minimum needed gene modules involved in the establishment of the *Arabidopsis* response to *T. urticae*. As expected, a high representation of genes related to response to a stimulus, in particular to jasmonic acid, was found. Further, a large number of genes were found with a potential role in the regulation of the transcription process, such as transcription factors and transcriptional regulators linked to the ubiquitination pathway.

### Data selection and analysis of *Arabidopsis* transcriptomes in response to herbivores

**Figure 3:**
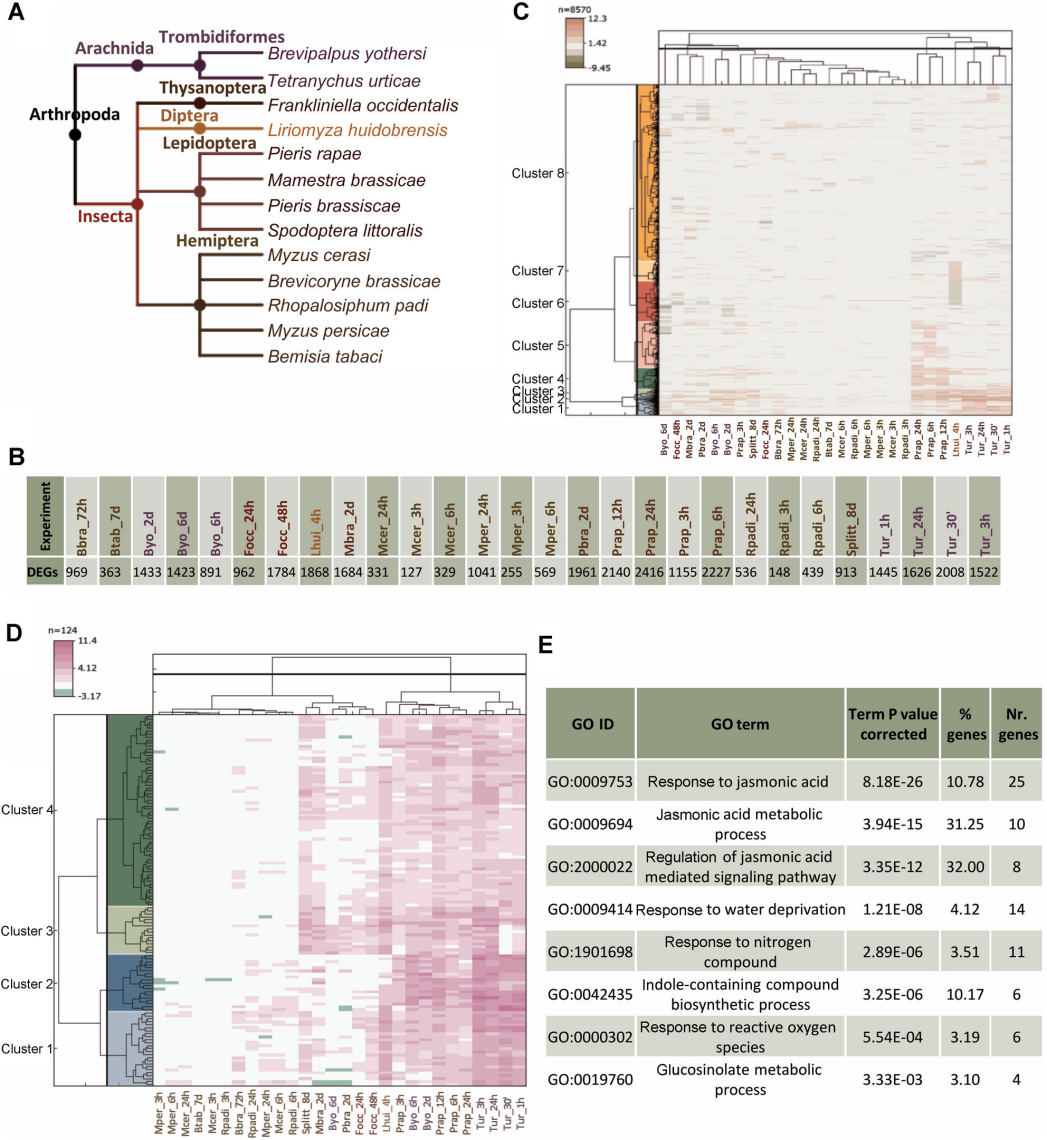
**Meta‐analysis of transcriptomic experiments showing the response of *Arabidopsis* plants to herbivore stresses** (**A**) Cladogram showing the taxonomic classification of the herbivore species. (**B**) Number of differentially expressed genes (DEGs) per experiment. (**C**) Heatmap showing the transcriptomic profile of all the DEGs at least detected in one experiment. (**D**) Heatmap showing the transcriptomic profile of the genes with a significant correlation among experiments. (**E**) Enriched biological processes associated with the correlated genes.

To get additional data useful for increasing the knowledge of the response of *Arabidopsis* to *T. urticae*, we extracted an initial collection of microarrays and RNA‐seq experiments regarding transcriptomic analyses of *Arabidopsis* plants exposed to different arthropod herbivores. Because of the high variability of the experimental conditions across the transcriptomic experiments, a subset of them was selected to extract more robust conclusions. To be comparable with our RNA‐seq experiment, all the selected experiments used *Arabidopsis* plants of the ecotype Columbia‐0 (Col‐0), with a preferably vegetative stage of 4 weeks when infested. A summary of the final list of selected experiments is shown in Table S2. The final collection was composed of 28 experiments, 17 with microarray data and 11 with RNA‐seq data. Experiments included different herbivores: lepidopterans, mites, aphids, leafminers, thrips, and hemipteran (Figure 3A). Most experiments used foliar tissue from 4‐week‐old plants. After this initial selection, DEGs were obtained for each experiment. Only data from the experiments of *Liriomyza huidobrensis*, *Brevicoryne brassicae*, and *Frankliniella occidentalis* insects needed to be re‐analyzed, as processed data were available for the rest of the experiments. Normalization was performed to reduce the non‐biological variability. The final list of DEGs showed high variability in the number of genes detected (Figure 3B). The number of DEGs varies from 127 in the case of the response to *Myzus cerasi* at 3 h to 2,416 in the case of the response to *Pieris rapae* at 24 h.

### Gene Ontology term enrichment analysis

Once the experiments were selected, the lists of DEGs were compared to determine the similarities or differences present among them (Data S4). For this purpose, a heatmap with the DEGs and experiments was generated (Figure 3C). The obtained results pointed out the existence of high variability in the plant response. None of the DEGs was differentially expressed in all the experiments, but several groups of DEGs were identified in the response to different species. For example, *L. huidobrensis* shared a partial common gene induction with *T. urticae* and *P. rapae*. Nevertheless, as the number of total DEGs was very large, further approaches were done to compare the modified gene expression among experiments.

As a first step to discover similarities in the global responses, the correlation in the expression of the genes that were differentially expressed in at least 10 experiments was analyzed (Data S5). From the 188 genes with any correlation, 124 showed a correlated expression with another gene of the same fully connected subnetwork. When a heatmap with the expression of these genes was performed, a robust pattern was detected (Figure 3D). Correlation results were characterized by a general absence of altered expression in aphids accompanied to a common up‐regulation for most genes in the other species. This module of coexpressed genes was mainly enriched in terms of biological processes associated with the jasmonic acid response and the metabolism of indole glucosinolates (Figure 3E).

### Clustering of experiments

**Figure 4:**
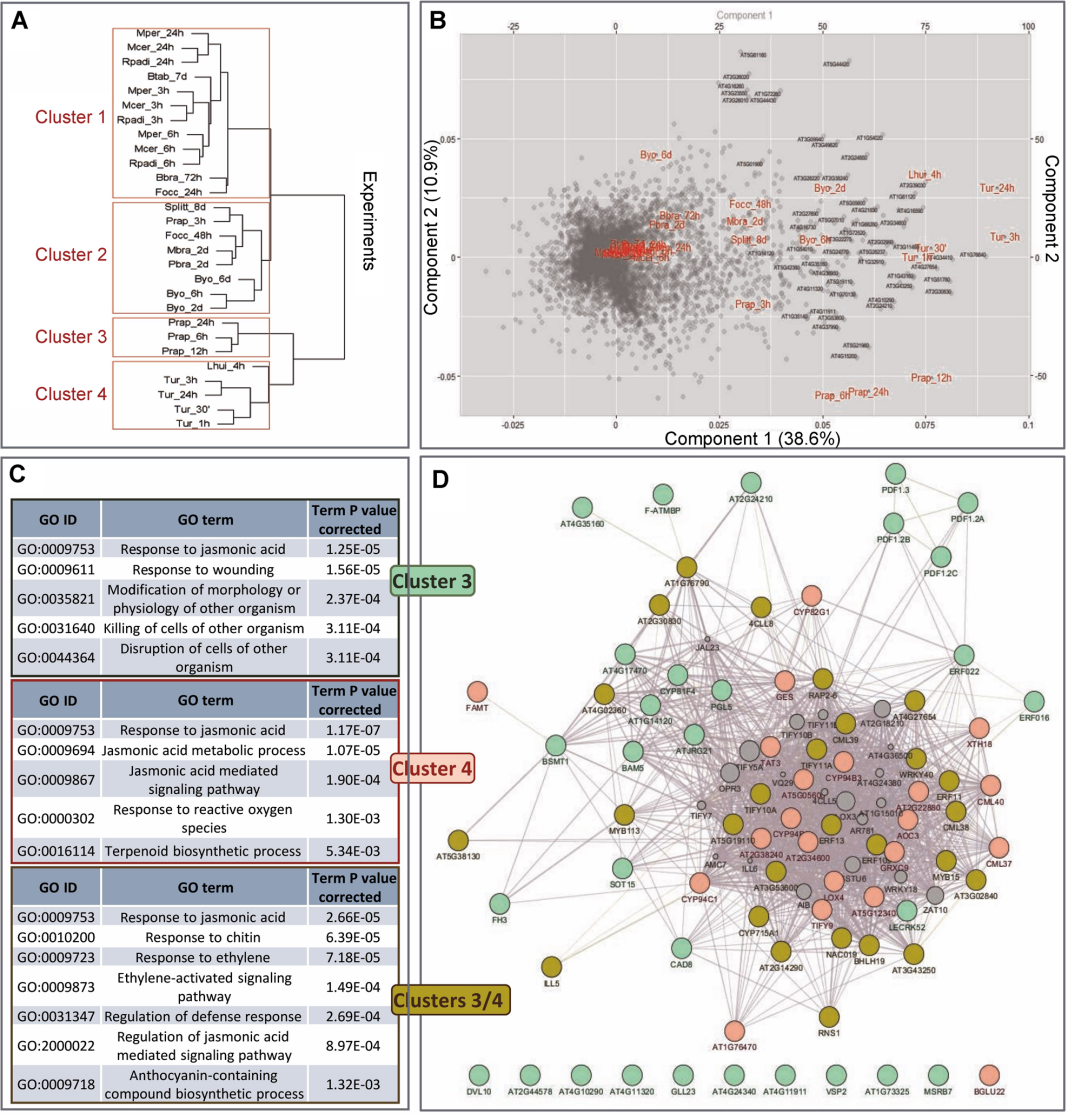
**Clustering of experiments and differentially expressed genes (DEGs)** (**A**) Hierarchical clustering of experiments based on the expression of the DEGs. (**B**) Biplot showing the distribution of the DEGs and experiments. Dots represent the genes in the biplot, and red color the experiments. Gene names of the top 100 genes with the highest contribution to the variability of the data are included. (**C**) Enriched biological processes associated with the genes induced specifically in any cluster. (**D**) Gene network connecting the DEGs induced specifically in cluster 3 (pale green), cluster 4 (pink), or both clusters (olive green). Size of additional nodes (gray) is based on the GeneMANIA scores.

Differential expression patterns shown in the heatmaps were quite compatible with an accurate clustering. For that reason, hierarchical clustering of the expression patterns was performed to determine which plant responses were more similar to each other. The four clusters option was selected for the hierarchical clustering (Figure 4A). In cluster 1, all the experiments including aphids were grouped. Further, the experiments performed with the silverleaf whitefly *Bemisia tabaci* and with the thrip *F. occidentalis* at 24 h were also included. Cluster 2 was constituted by experiments with the lepidopterans *Pieris brassicae*, *Mamestra brassicae*, *Spodoptera littoralis* and *P. rapae* at 3 h, the thrip *F. occidentalis* at 48 h, and the mite *Brevipalpus yothersi*. In cluster 3, only the experiments performed with *P. rapae* at 6, 12, and 24 h were included. Finally, in cluster 4, the experiments with the spider mite *T. urticae* and the leafminer *L. huidobrensis* were grouped.

### Contribution of experiments and DEGs to clustering

Principal component analysis was performed for visualizing the underlying relationships between the experiments and the DEGs in them. Experiments were used as the source of variation of the DEGs, trying to find those genes that have a special profile of expression through the experiments. These genes would be the most relevant to discriminate among the transcriptomic responses. For this purpose, a PCA biplot was depicted, showing both PCA scores plot with the gene classification and the PCA loading plot, with the weight of the experiments on the PCA (Figure 4B). The PCA plot showed a quite high number of genes located near the origin and a low number of them far from it. In the case of the experiments, considering the 49.5% of the variability of the data explained by the two first components, the experiments performed with *T. urticae*, *L. huidobrensis* and *P. rapae* exhibited the most different transcriptomic response. Locations in the plot displayed notable similarities with the clustering classification, being the experiments of each cluster located near to each other in the plot. Therefore, according to the biplot, those genes in which classification is strongly positive in component 1 are more likely to be important in the transcriptomic response of the plant to *T. urticae*, *L. huidobrensis* and *P. rapae*. Further, if those genes also have a strongly negative value in component 2, they are more likely relevant genes in the transcriptomic response to *P. rapae*.

To obtain a higher precision in the analysis, the dimensions explaining the majority of the variability of the PCA were selected (Figure S3A). Then, the experiments and top 100 genes with the highest contribution to these dimensions were extracted, being those genes relevant in the *Arabidopsis* response to these experiments (Figure S3B, C; Data S6). The experiments with the highest contribution in the eight first dimensions of the PCA were those experiments performed with *L. huidobrensis*, *T. urticae* and *P. rapae*. This information was similar to that observed in the PCA biplot. The identifiers of the top 100 genes were also plotted in the PCA biplot to mark their position. All of them showed a prominent contribution in the first PCA dimension, being located on the right part of the biplot. To elucidate their relevance and specificity, an analysis of the differential expression of these 100 DEGs across the four clusters was performed (Figure S4A). The number of relevant DEGs in each cluster and their intersections were calculated (Figure S4B, C). Clusters 3 and 4 contained the highest numbers of genes with confidence intervals significantly higher than the log_2_FC mean, many of them shared by both clusters. The individual genes significantly deregulated in clusters 3 and 4 are compiled in Table S3. Three groups of genes could be found, those with significantly higher expression in cluster 4 (20 genes), clusters 3 and 4 (27 genes), or cluster 3 (26 genes). Also, four genes in cluster 3 had a significantly lower expression. Enriched biological processes for the three groups are mainly related to the jasmonic acid signaling pathway, with some particularities (Figure 4C). Cluster 3 is enriched in genes associated with a direct killing response, cluster 4 in genes leading to the biosynthesis of terpenoids, and several shared induced genes in both clusters are related to the ethylene signaling pathway and the biosynthesis of anthocyanins. When a network was built using the GeneMANIA tool in Cytoscape, most genes from the three groups were connected (Figure 4D). However, whereas genes from cluster 4 had a predominant central position in the network, genes from cluster 3 were predominantly located at the periphery of the network or were unconnected with the rest of the nodes.

### Specificities in the response to *T. urticae*

**Figure 5:**
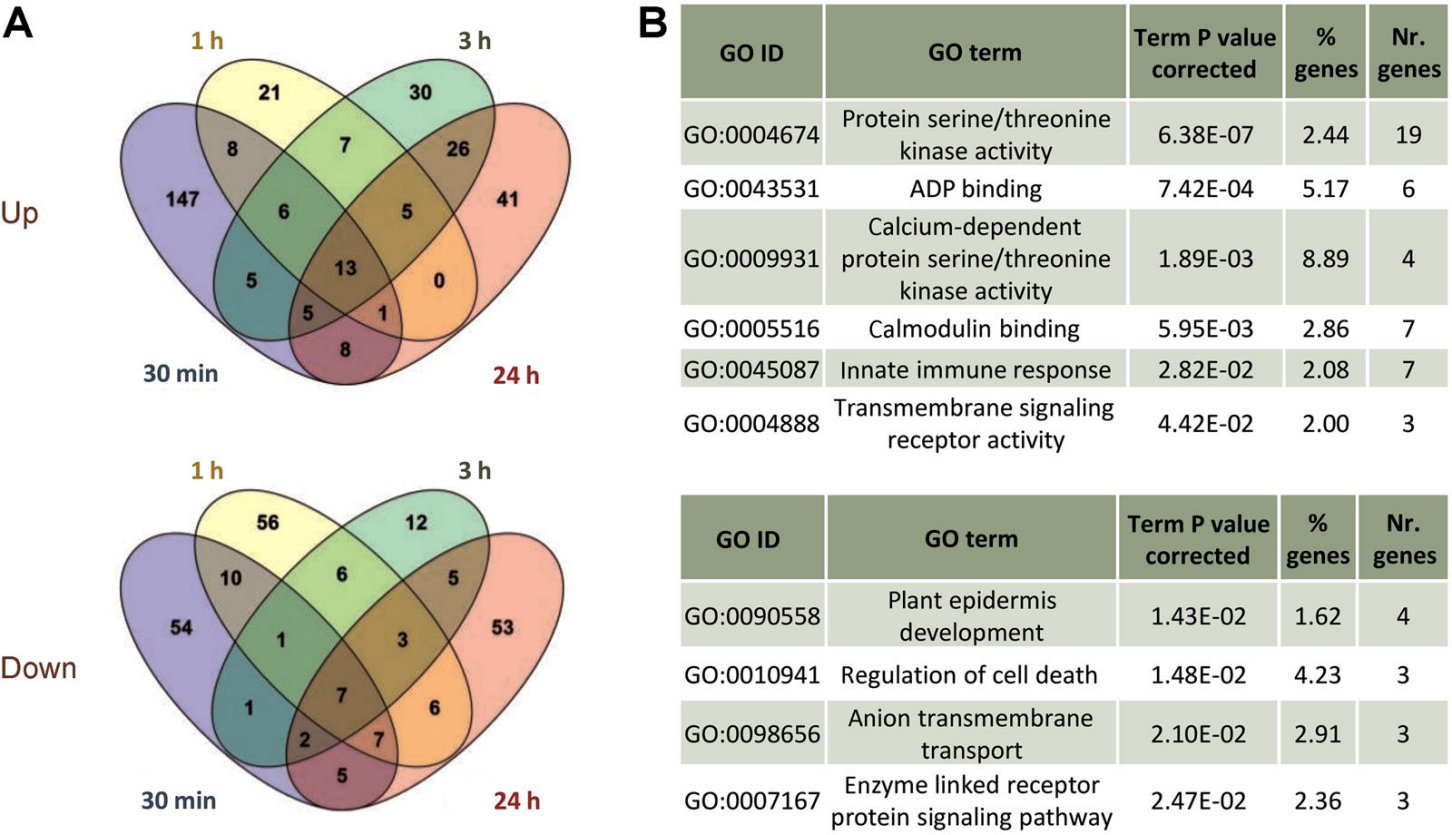
**Analysis of differentially expressed genes (DEGs) only detected upon *Tetranychus urticae* infestation** (**A**) Venn diagrams showing the number of specific and shared up‐ or down‐regulated genes. (**B**) Enriched biological processes and molecular functions in the specific genes up‐regulated at 30 min or down‐regulated at 30 min or 1 h of infestation.

As the response of the plant to the different herbivores showed a broad range of putative similarities and specificities, the DEGs only found in the *T. urticae* experiments were analyzed (Data S7). Among these *T. urticae* specific genes under the present data, 323 up‐regulated and 228 down‐regulated, many DEGs were time point specific (Figure 5A; Data S8). The highest number of up‐regulated genes was found at 30 min upon infestation and 30 min, 1, and 24 h time points shared similar numbers of down‐regulated genes. Interestingly, whereas up‐regulated genes at 30 min were enriched in biological processes related to the perception and first steps of signaling, the first biological process enriched in the down‐regulated genes at 30 min and 1 h was the plant epidermis development (Figure 5B).

**Figure 6:**
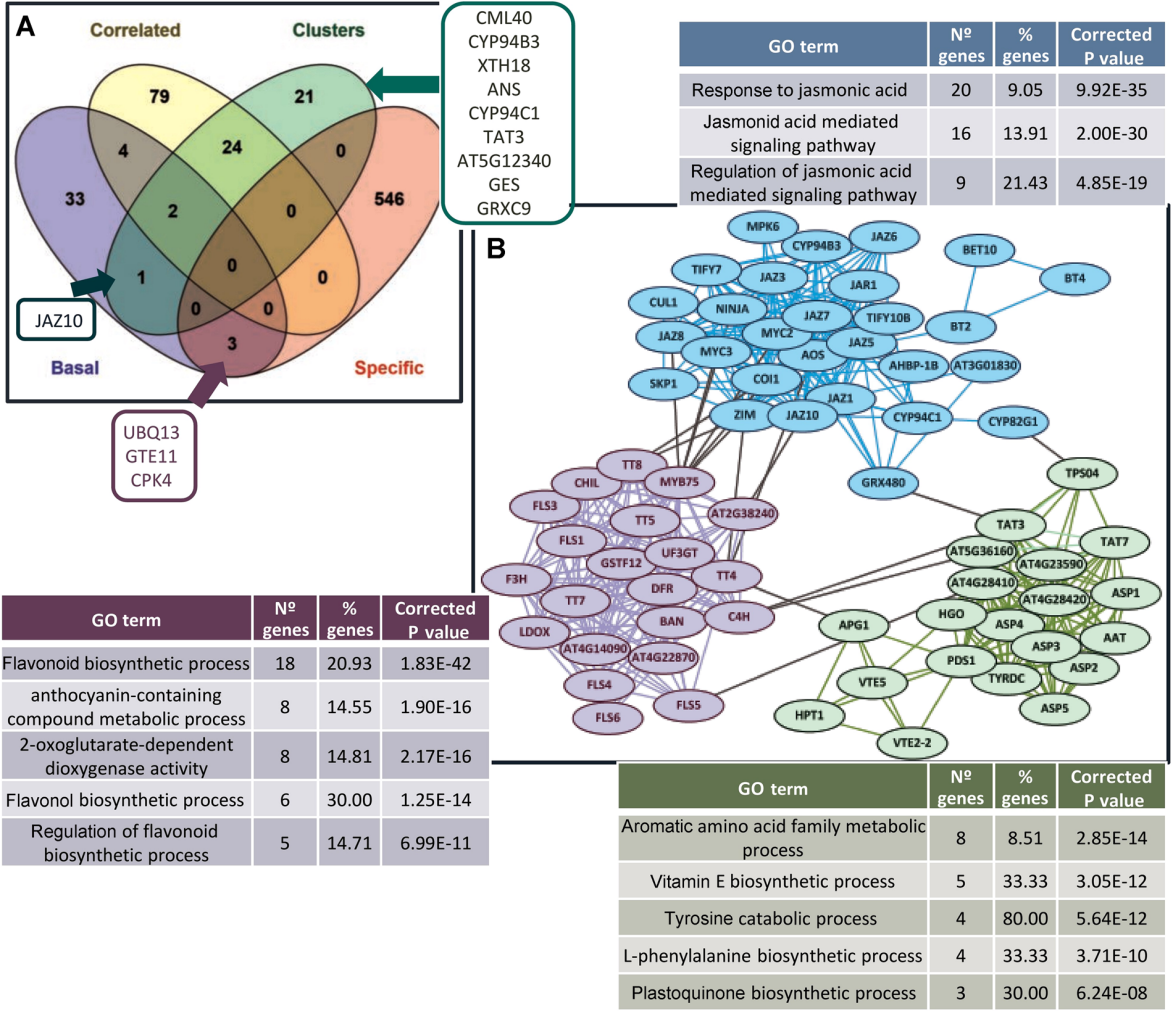
**Prediction of the *Arabidopsis* enhanced responses to *Tetranychus urticae* infestation** (**A**) Venn diagram comparing the genes involved in the basal network constructed by spider mite RNA‐seq data, the genes in which expression was correlated among herbivore experiments, the genes with a significant contribution to the cluster with the *T. urticae* experiments, and the genes specifically deregulated upon spider mite infestation. (**B**) STRING‐based network after eight rounds of adding nodes to an initial set composed of the three specific genes that appeared in the basal network, the gene shared by the basal and cluster gene sets, and the nine genes not correlated that were significantly different only in the cluster with the *T. urticae* experiments. Enriched biological processes for the three putative subgroups of the network are included.

Finally, many of the results previously obtained were integrated to establish a preliminary model on the key biological processes involved in the response of the plant to the spider mite infestation. A comparison was performed between: (i) the genes involved in the basal network constructed by the spider mite RNA‐seq data (Figure 2); (ii) the genes in which expression was correlated among herbivore experiments (Figure 3); (iii) the genes with a significant contribution to separate the cluster with *T. urticae* experiments from the rest of the clusters (Figure 4); and (iv) the genes only found deregulated upon spider mite infestation (Figure 5). The Venn diagram showed that only three out of 549 genes specifically deregulated in *T. urticae* and six of the correlated genes appeared among the 43 genes used to construct the basal network (Figure 6A; Data S9). Further, many of the genes contributing to the spider mite cluster classification showed a correlation among experiments and only one gene was also present in the basal network. From these results, a STRING‐based network was built after six rounds of adding nodes to an initial set composed of the three specific genes that appeared in the basal network, the gene shared by the basal and cluster gene sets, and the nine genes that were uncorrelated among experiments and were significantly different from all the other clusters (Figure 6B). Three clusters of functionally related genes were obtained. Enrichment of the biological processes involved showed an expected defensive response related to the signal transduction mediated by jasmonic acid. Further, several metabolic pathways were enriched, such as those related to the production of flavonols and anthocyanins, the metabolism of aromatic amino acids, and the synthesis of tocopherols.

## DISCUSSION

One of the major challenges in plant biology is to understand how plants rewire their molecular machinery to cope adequately with abiotic/biotic stresses. Here, we aimed to elucidate the usefulness of comprehensive meta‐analysis to discover hidden gaps not covered by individual plant transcriptomics responses to herbivores. For that, we took advantage of the bioinformatics tools and expression databases publicly available. Despite inherent variability, a core of pathways triggered by the main stress may be found. In *Arabidopsis*, the Bla‐2 accession is more resistant to the attack of *T. urticae* than the Kondara accession, but their responses are commonly based on the induction of the jasmonic acid hormonal pathway and the production of indole glucosinolate (IG) metabolites (Zhurov et al., 2014). Likewise, induction of the metabolic pathway for IG production was found in 19 *Arabidopsis* accessions upon infestation with different insects, although differences in the up‐regulated genes were reported (Sato et al., 2019). Therefore, useful findings may be obtained from an *in silico* analysis of both, the time course experiment with *T. urticae* and the meta‐analysis comparing transcriptomic data of the *Arabidopsis* response to different arthropod herbivores. Dissected information from individual modules is required to establish a final molecular model.

### Module 1. Dissecting information from the time course experiment

Early responses upon 1 h spider mite infestation were previously described as involved in signaling and regulation of gene expression and mostly maintained until 24 h infestation (Zhurov et al., 2014). More specific information arises on the first regulatory steps concerning gene expression by taking an earlier time point. In fact, a substantial number of genes with a rapid and transient up‐ and down‐regulation appeared upon 30 min mite infestation. As expected, this set of genes was enriched in both extra and intracellular receptors, in genes related to signaling by calcium levels and kinase/phosphatase activities, and in genes involved in the vesicular transport of proteins.

To unravel the meaning of extensive changes in gene expression, the information coming from the differential expression analyses must be properly processed. Enrichment of Gene Ontology terms and network analyses are useful to establish key processes and molecular connections. Jasmonic acid signaling and IG production were pointed out as the most remarkable events in the response of *Arabidopsis* to the spider mite, with a certain role for salicylic acid (SA) signaling (Zhurov et al., 2014). As expected, these biological processes were enriched in our set of data along the time course. Network algorithms based on protein‐protein interactions have been postulated as key tools to adequately provide a systems view of plant defense (Windram et al., 2014). In the present analysis, a large number of individual DEGs became connected when the NetworkAnalyst program was applied. Therefore, a snapshot of the interactions between proteins at different time points arises. These interactions permit the discovery of important genes connecting functionally related modules that were not up‐ or down‐regulated upon a mite attack. Nodes with high connectivity and betweenness are enriched in conditional phenotypes and are positively related to the interaction with pathogen effectors and the modulation of plant immunity (Ahmed et al., 2018). These nodes are called hubs, defined as the highest connected central proteins in scale‐free protein‐protein interaction networks (Vandereyken et al., 2018). Our centrality measures detected the TF MYC2, and the ubiquitin‐related proteins UBQ13 and BT2 as putative hubs connecting functional modules along with the response of *Arabidopsis* plants to spider mites. The relevance of MYC2 in the response to herbivory has been broadly documented, with a crucial role in the signaling pathway activated by jasmonic acid (Kazan and Manners, 2013). BT2 has been proposed as an essential component connecting and integrating multiple signaling routes, including the jasmonic acid pathway (Mandadi et al., 2009), and was identified as the most central element of the nitrogen use efficiency molecular network (Araus et al., 2016). These central features of BT2 may be explained by its scaffolding role. BT2 binds to calmodulin and interacts with CUL3 forming an E3 ubiquitin‐protein ligase complex and with the general transcription activators GTE9 and GTE11 (Du and Poovaiah, 2004; Figueroa et al., 2005; Gingerich et al., 2005). Finally, *UBQ13* is a ubiquitin‐encoding gene, and the ubiquitin system has been described as a signaling hub for the integration of environmental signals (Miricescu et al., 2018).

Starting from these three central proteins, the network obtained by adding interacting proteins to connect them could be established as a basal frame of the molecular plant response to the spider mite. As expected, this core frame englobes the response to a stimulus associated with the jasmonic acid signaling pathway and the regulatory mechanisms carried out by TFs and the ubiquitination system. Connections between nodes reflect the relevance of the three hub proteins and their interactions with other key proteins. For example, the interactions of MYC2 with the GA‐related protein RGL3 or the cytokinin‐signaling regulator AHP5 reflect the previously reported participation of MYC2 in the crosstalk of jasmonic acid with other hormonal signaling pathways (Jang et al., 2020). PUB10 and TIC are two proteins that negatively regulate MYC2 by ubiquitination or repression (Shin et al., 2012; Jung et al., 2015). Finally, several MYB transcription factors (TFs) are connected to MYC2 in the network. Members of the MYB and bHLH TF families act together regulating the biosynthesis of secondary metabolites. MYB21 interacts with MYC2 to control the expression of terpene synthase genes (Yang et al., 2020); MYB28, 29, 34, and 122 interact with MYC2, both being involved in the production of indole and aliphatic glucosinolates (Schweizer et al., 2013); and MYB24 together with a set of MYB‐bHLH interacting partners regulate the biosynthesis of flavonoids and anthocyanins (Xu et al., 2014; Battat et al., 2019).

### Module 2. Dissecting information from the transcriptomic meta‐analysis

Individual information on one species may be significantly enriched with data from related species. The ultimate goal is to increase knowledge on several issues related to individual data, like the specificity in the response of the plant to a herbivore or the plant response patterns common to arthropod herbivores feeding on *Arabidopsis*. The observed specificity in the response was strikingly high. Regarding the two RNA‐seq experiments with four times‐associated samples, more than 500 genes were exclusively deregulated upon *T. urticae* infestation, a number that was even higher upon *P. rapae* infestation, nearly 900 genes. In a previous large‐scale transcriptome analysis in *Arabidopsis* based on microarray data from 14 pathogen species, more than 25% of deregulated genes were species‐specific (Jiang et al., 2017), confirming the enormous plasticity of the *Arabidopsis* response.

However, most responses trigger a set of common signaling pathways related to plant defense. The set of genes with a correlated expression in response to herbivores was enriched in genes related to jasmonate and nitrogen compound response or glucosinolate metabolism. These categories have been broadly associated with biotic stresses. As broadly known, jasmonic acid signaling is a conserved core pathway in herbivore‐induced responses (Wang et al., 2019). The production of IG occurs in response to many biotic attacks, being secondary metabolites toxic to a broad range of microorganisms, nematodes and insects (Wittstock and Burow, 2010). Likewise, the chitin of phytopathogenic fungi, nematodes and arthropods is recognized by the plant, activating innate or adaptive plant defense responses (Jiang et al., 2019). These features support that the identified set of genes rightfully belongs to a basal signaling pathway triggered by herbivory. This common pathway would be modulated by additional inputs coming from the specificities in the perception of each herbivore species. Inputs are directly related to outputs. Our analysis clearly points to the association of several genes to specific responses. The expression patterns showed by these genes strongly build the most robust gene‐cluster associations, which were found in the responses against *P. rapae* or the cluster formed by *T. urticae* and *L. huidobrensis*. Similarities between *T. urticae* and *L. huidobrensis* experiments suggest a common recognition and defense response by the plant, which could be in some way explained by their feeding features. Both species feed on the palisade and spongy mesophyll of the leaf causing cell death only of the consumed cells (Bensoussan et al., 2016; Weintraub et al., 2017).

### Module 3. Compiling information to discover the underlying key molecular aspects

Virtually, mining of transcriptomes and secondary analyses should offer realistic clues on the particularities involving the plant response in an individual plant‐herbivore interaction. However, there is not an optimal way to deal with the analysis of meta‐transcriptomes due to the variability in the approaches and conditions used in the correction of experimental bias and the subjective interpretations of integrated data. Consequently, an intuitive assay based on the previous results of analysis emerges as the sub‐optimal method to extract conclusions. Although uncertainties are likely found, this kind of analysis entails substantial contributions to robust species‐based studies. In a first attempt to disentangle the principal features of the *Arabidopsis* response to *T. urticae* infestation, several considerations were taken into account to combine the transcriptomic analysis of the response to *T. urticae* with the transcriptomic meta‐analysis of *Arabidopsis* responses to arthropod herbivores.

First, jasmonic acid signaling, response to chitin, and glucosinolate metabolism represent the master responses against herbivores in *Arabidopsis* plants. As these processes are the most enriched by the genes with a correlated expression among experiments, they could be included in the set of regulated genes with a low species specificity. Second, the predicted basal network for the *Arabidopsis* response to *T. urticae* comes from a previous selection of the most probable genes acting as hubs derived from the individual response to *T. urticae* and could be involved in the specificity of the response. Third, an elevated number of genes was only detected as deregulated upon *T. urticae* infestation, which could equally be involved in the particular pathways triggered by *T. urticae*. Fourth, the genes that are significantly contributing to the clustering of experiments have a reasonable probability to be involved in specific rewires in the transcriptional response. Thus, the 10 genes significantly more expressed in the cluster with the *T. urticae* experiments that were uncorrelated, and the three genes included in the predicted basal network and specifically induced by *T. urticae* are the best candidates to participate in the enhanced responses triggered by the specific perception associated with *T. urticae* herbivory.

Interestingly, these likely essential mite‐regulated genes connect jasmonate and defensive responses with metabolic pathways leading to the production of anthocyanin‐containing compounds and terpenoid‐related metabolites. Connections between jasmonic acid response and anthocyanin synthesis are mediated by MYC2 and the induction of specific WD‐repeat/bHLH/MYB modules (Xu et al., 2014). These specific TFs, like *PAP1* (*MYB75*), and the bHLH genes *TT8* and *GL3* were up‐regulated upon mite infestation. As expected, these activated complexes led to the up‐regulation of several enzymes involved in the biosynthesis of anthocyanins, such as the dihydroflavonol reductase DFRA, the leucoanthocyanidin dioxygenases/anthocyanidin synthases LDOX and ANS, or the anthocyanin glucosyltransferases UGT75C1 and UGT79B1 (Saito et al., 2013). Further, two specific pathways in the biosynthesis of terpenoid‐related metabolites appeared as de‐regulated. The first route leads to the synthesis of the herbivore‐induced volatile C16‐homoterpene TMTT (E,E‐4,8,12‐trimethyltrideca‐1,3,7,11‐tetraene) from GGPP (geranylgeranyl diphosphate). TMTT influenced the foraging behavior of predatory mites when emitted from lima bean leaves infested by spider mites (de Boer et al., 2004). The enzymes involved in the two steps of the route, TPS04/GES and CYP82G1, were induced upon *T. urticae* attack, supporting a relevant role for TMTT in the *Arabidopsis* response. The second route connects tyrosine metabolism with tocopherol production. A key enzyme is the mite‐induced tyrosine aminotransferase TAT3, which was previously shown to be up‐regulated by wounding and jasmonic acid (Yan et al., 2007). TAT3 catalyzes the reversible transamination from tyrosine to form 4‐hydroxyphenylpyruvic acid (pHPP). pHPP can be converted to homogentisic acid, the aromatic precursor of tocopherols and plastoquinone. Tocopherols have been associated in *Arabidopsis* to an effective basal resistance against compatible *Pseudomonas syringae* and the activation of defenses when challenged with *Botrytis cinerea* (Cela et al., 2018; Stahl et al., 2019). The up‐regulation of HPT1, APG1, and VTE2‐2, enzymes involved in the biosynthesis of tocopherols from homogentisic acid, supports an undescribed relevant role of these compounds in the coordinated response to *T. urticae* infestation.

In conclusion, the combination of our own transcriptomic data with data from public repositories enables us to reasonably predict novel relevant processes and specificities involved in the *Arabidopsis* response to the spider mite. Thus, dual individual/general analysis of the transcriptomic responses should be considered a robust tool to be integrated into biotechnological projects. In the next few years, new data from RNA‐seq experiments and novel bioinformatics tools will allow the construction of more robust databases and to perform better analyses. As a consequence, it is expected there will be an exceptional generation of knowledge on how crops recognize and respond to different biotic agents.

## MATERIAL AND METHODS

### Plant material and growth conditions

*Arabidopsis thaliana* L. Col‐0 accession was used. Seeds were planted and incubated 5 d in the dark at 4°C in autoclaved peat moss and vermiculite (3:2 V/V). Plants were then grown in growth chambers (Sanyo MLR‐350‐H) under control conditions (23°C ± 1°C, >70% relative humidity, and a 16 h/8 h day/night photoperiod).

### Spider mite maintenance and plant infestation

A colony of *T. urticae*, London strain (Acari: Tetranychidae) provided by Dr. Miodrag Grbic (UWO, Canada), was reared on beans (*Phaseolus vulgaris*) and maintained in growth chambers (Sanyo MLR‐350‐H) at 25°C ± 1°C, >70% relative humidity and a 16 h/8 h day/night photoperiod. Three‐week‐old plants were infested with 20 *T. urticae* female adults per plant. They were carefully transferred with a brush to the leaf surface. Plant material was harvested after 0 h, 30 min, 1, 3, and 24 h of infestation.

### RNA‐seq library preparation, sequencing, alignment, and DEG analysis

Total RNA was isolated and purified by using RNeasy Qiagen Mini Plant Kit (74904 Qiagen), including the on‐column DNA I (79254, Qiagen) digestion recommended by the manufacturer. RNA amount and quality were tested in a Nanodrop ND‐1000. Total RNA was sent to Centre for Genomic Regulation (CNAG‐CRG) (Barcelona, Spain). Double‐stranded cDNA libraries obtained from purified mRNA were sequenced using Illumina HiSeq™ 2000 high throughput sequencing technology. More than 40 M paired‐end reads were obtained for each sample (*n* = 3). Three biological replicates coming from three independent experiments were used. For each biological replicate, six rosettes were pooled and frozen in liquid nitrogen. Reads were mapped to the *Arabidopsis* reference genome (ensemble release 39, TAIR10) using STAR aligner version 2.5.3a (Dobin et al., 2013) with ENCODE standard options for long RNA‐seq. Mapped reads were quantified at “Gene” level with RSEM version 1.3.0 with default parameters (Li and Dewey, 2011). Differential expression analysis was performed with DESeq2 version 1.18 (Love et al., 2014) with default settings. Size factors calculation and dispersion estimation were done with samples from all time points together. For hypothesis testing the Wald test with the “contrast” function was used to compare groups of interest (always using time 0 as the reference group). Differentially expressed genes were considered those genes showing a p‐adjusted value <0.05 and a log_2_Ratio (fold change) higher than 1. Venn diagrams were performed using the Venny 2.1 tool (Oliveros, J.C., 2007–2015, https://bioinfogp.cnb.csic.es/tools/venny/index.html). Gene enrichment analyses were performed with the Bonferroni step‐down test using ClueGO package (Bindea et al., 2009) in Cytoscape (Shannon et al., 2013). Comparison of total DEGs across selected experiments was conducted using Instant Clue software (Nolte et al., 2018), which perform a hierarchical clustering to classify the experiments and generate a heatmap for the visualization of the similar patterns of DEGs. The datasets generated during the current study are available in the ArrayExpress repository, accession number E‐MTAB‐9448. Real‐time RT‐qPCR analysis for expression comparisons is described in Supporting Methods.

### Searches in transcriptomic databases and analysis of selected experiments

To examine the transcriptomic responses to biotic stresses mediated by phytophagous arthropods, we searched in different public repositories of gene expression patterns under diverse biotic stress conditions. Microarrays and RNA‐seq experiments were collected from Expression Atlas (http://www.ebi.ac.uk/gxa), ArrayExpress (https://www.ebi.ac.uk/arrayexpress) or National Center for Biotechnology Information Gene Expression Omnibus (https://www.ncbi.nlm.nih.gov/geo/), using “transcriptomics”, “biotic stress”, “*Arabidopsis*” or “herbivore” as keywords with no restrictions in the date of publication. Transgenic and resistant genotypes were excluded from the analysis. Analysis of microarray and RNA‐seq data are described in Supporting Methods. Comparisons of DEGs across selected experiments were conducted using Instant Clue software (Nolte et al., 2018). The ExpressionCorrelation plugin for Cytoscape (http://www.baderlab.org/Software/ExpressionCorrelation) was used to identify correlated genes among experiments. The similarity matrix was computed using a threshold of 0.95 for the Pearson correlation coefficient.

### Clustering and analysis of experiments and DEGs

To understand the similarities and differences in the response of *A. thaliana* to different herbivores, hierarchical clustering of the DEG lists was performed. To that end, Euclidean distances were calculated following Ward's linkage method with the hclust function in the “stats” package of R (v.3.5.2). Principal component analysis was also performed using the princomp function in R software (v.3.5.2). To examine the classification of DEGs and the underlying relationship between experiments and DEGs, eigenvalues and contributions were calculated. Experiments were used as variables to see which genes have a specific or peculiar behavior in response to specific experiments. Based on the explanation of over 70% of the total variation and the presence of an inflection point in the scree plot, the most important dimensions for the analysis were selected (Cattell, 1966). Using the previous information, the experiments with the highest contribution to the variability of the expression of the genes and the top 100 DEGs that respond more specifically to this variation in these dimensions were extracted. Analysis of their expression through the clusters previously generated was developed to identify which DEGs were more relevant. Because of the nature of the data, bootstrapped non‐parametric bias‐corrected and accelerated (BCa) confidence intervals of the log_2_FC for each DEG in each cluster were calculated with the percentiles 0.025 and 0.0975 and 10,000 replications using the boot.BCa function in R. DEGs in a cluster were considered to have a relevant behavior in a cluster when the mean value of the log_2_FC of all genes through all experiments was not included within their bootstrapped confidence interval.

### Molecular networks

Several available tools were used to construct gene molecular networks. NetworkAnalyst is a platform that builds molecular networks based on the qualitative expression of a gene and the protein‐protein interactions generated in the STRING database version 11.0 (Szklarczyk et al., 2019; Zhou et al., 2019). Using the confidence score higher than 900 and experimental evidence required as parameters and the minimum connected network option, the significant genes are mapped to the corresponding molecular interaction database. Further, the GeneMANIA tool for Cytoscape (Montojo et al., 2010) and the own STRING database were selected to construct protein‐protein interaction networks with increasing complexity.

## AUTHOR CONTRIBUTIONS

I.D. and M.M. conceived the research. M.E.S., A.G., A.A., I.R.D., and G.R.H. performed the experimental research. All authors contributed to the final version of the manuscript.

## Supporting information

Additional Supporting Information may be found online in the supporting information tab for this article: http://onlinelibrary.wiley.com/doi/10.1111/jipb.13026/suppinfo

**Data S1.** Differentially expressed genes in *Arabidopsis* at four times of mite infestation

**Data S2.** Lists of specific and shared up‐ or down‐regulated genes corresponding to Figure 1B

**Data S3.** List of genes in networks built from differentially expressed genes in *Arabidopsis* at four times of mite infestation

**Data S4.** Differentially expressed genes in *Arabidopsis* upon infestation using different herbivores

**Data S5.** Differentially expressed genes in *Arabidopsis* with a correlated expression upon infestation using different herbivores

**Data S6.** List of genes in clusters with a significant deviation from the mean value

**Data S7.** Differentially expressed genes specifically deregulated in *Arabidopsis* upon infestation with *Tetranychus urticae*

**Data S8.** Lists of specific and shared up‐ or down‐regulated genes corresponding to Figure 5A

**Data S9.** Lists of specific and shared regulated genes corresponding to Figure 6A

**Figure S1.** Analysis of RNA‐seq reads from *Arabidopsis* samples infested with *Tetranychus urticae* at 0 h, 30 min, 1, 3, and 24 h
(**A**) Principal component analysis (PCA) showing the distribution of the samples in the two first dimensions. (**B**) Percentage of mapped reads per chromosome. (**C**) Mapping statistics. (**D**) Number of genes detected with at least one mapping.
**Figure S2.** Linear graphics comparing the expression pattern of 15 selected genes by RNA‐seq and quantitative real‐time polymerase chain reaction techniques
**Figure S3.** Contribution of genes and experiments to principal component analysis (PCA) main dimensions
(**A**) Scree plot and percentage of variation explained by each PCA dimension. Red circle indicates the presence of a point of inflection. (**B**) Top 15 experiments and (**C**) top 100 genes with the highest contributions in the eight first PCA dimensions. Red dashed lines indicate the mean value of percentage contribution shown through all experiments or genes.
**Figure S4.** Analysis of confidence intervals for clustering‐responsible genes
(**A**) Representation per cluster of the bootstrapped confidence intervals against the log_2_FC mean from the top 100 clustering‐responsible genes. Confidence intervals significantly different from the log_2_FC mean for each cluster are colored in red. (**B**) Number of genes significantly different from the log_2_FC mean in each cluster. (**C**) Venn diagram showing the specific and shared significant genes for each cluster.
**Table S1.** Enriched biological processes upon 30 min, 1, 3, and 24 h of *Tetranychus urticae* infestation
**Table S2.** Selected transcriptomic experiments for the analysis of the response of plants to herbivore attack. Accession number of the experiment is provided as Gene Expression Datasets Series (GSE) from the Gene Expression Omnibus (GEO) platform, as E‐MTAB accession number from ArrayExpress database, as SRP accession number from the Sequence Read Archive (SRA) or as NASCarray experiments from the Nottingham Arabidopsis Stock Centre's microarray database. The presence of an asterisk (*) indicates that a re‐analysis of the data was performed
**Table S3.** Individual genes significantly deregulated in clusters 3 and 4
**Table S4.** Oligonucleotide sequences for quantitative real‐time polymerase chain reaction analysis
**Supporting Methods.** Real‐time quantitative real‐time polymerase chain reaction analysis and analyses of stored microarray and RNA‐seq data
