# Supplementary material for: Comparative transcriptomics reveals hidden issues in the plant response to arthropod herbivores
Source: J Integr Plant Biol. 2021 Feb 12;63(2):312–26. doi: 10.1111/jipb.13026 (PMC7898633; doi:10.1111/jipb.13026)
Supplement: Supplementary file 10 — Figure S1. Analysis of RNA‐seq reads from Arabidopsis samples infested with Tetranychus urticae at 0 h, 30 min, 1, 3, and 24 h (A) Principal component analysis (PCA) showing the distribution of the samples in the two first dimensions. (B) Percentage of mapped reads per chromosome. (C) Mapping statistics. (D) Number of genes detected with at least one mapping. Figure S2. Linear graphics comparing the expression pattern of 15 selected genes by RNA‐seq and quantitative real‐time polymerase chain reaction techniques Figure S3. Contribution of genes and experiments to principal component analysis (PCA) main dimensions (A) Scree plot and percentage of variation explained by each PCA dimension. Red circle indicates the presence of a point of inflection. (B) Top 15 experiments and (C) top 100 genes with the highest contributions in the eight first PCA dimensions. Red dashed lines indicate the mean value of percentage contribution shown through all experiments or genes. Figure S4. Analysis of confidence intervals for clustering‐responsible genes (A) Representation per cluster of the bootstrapped confidence intervals against the log2FC mean from the top 100 clustering‐responsible genes. Confidence intervals significantly different from the log2FC mean for each cluster are colored in red. (B) Number of genes significantly different from the log2FC mean in each cluster. (C) Venn diagram showing the specific and shared significant genes for each cluster. Table S1. Enriched biological processes upon 30 min, 1, 3, and 24 h of Tetranychus urticae infestation Table S2. Selected transcriptomic experiments for the analysis of the response of plants to herbivore attack. Accession number of the experiment is provided as Gene Expression Datasets Series (GSE) from the Gene Expression Omnibus (GEO) platform, as E‐MTAB accession number from ArrayExpress database, as SRP accession number from the Sequence Read Archive (SRA) or as NASCarray experiments from the Nottingham Arabidopsis Stock Ce [file JIPB-63-312-s010.pdf]

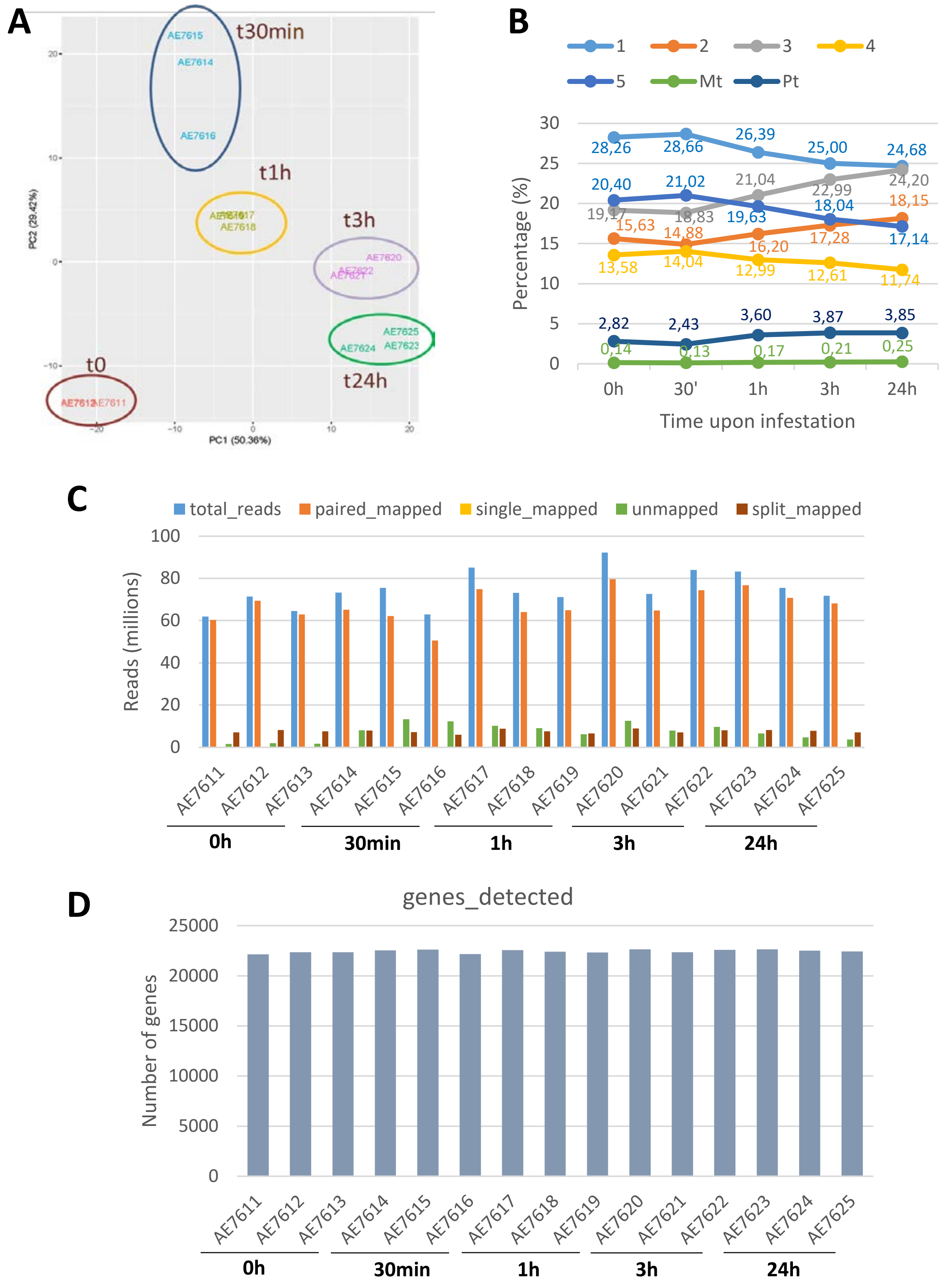

Figure S1. Analysis of reads from Arabidopsis samples infested with *T. urticae* during 0h (AE7611-13), 30min (AE7614-16), 1h (AE7617-19), 3h (AE7620-22), and 24h (AE7623-25). **(A)** Principal Component Analysis (PCA) showing the distribution of the samples in the two first dimensions. **(B)** Percentage of mapped reads per chromosome. **(C)** Mapping statistics. **(D)** Number of genes detected with at least one mapping.

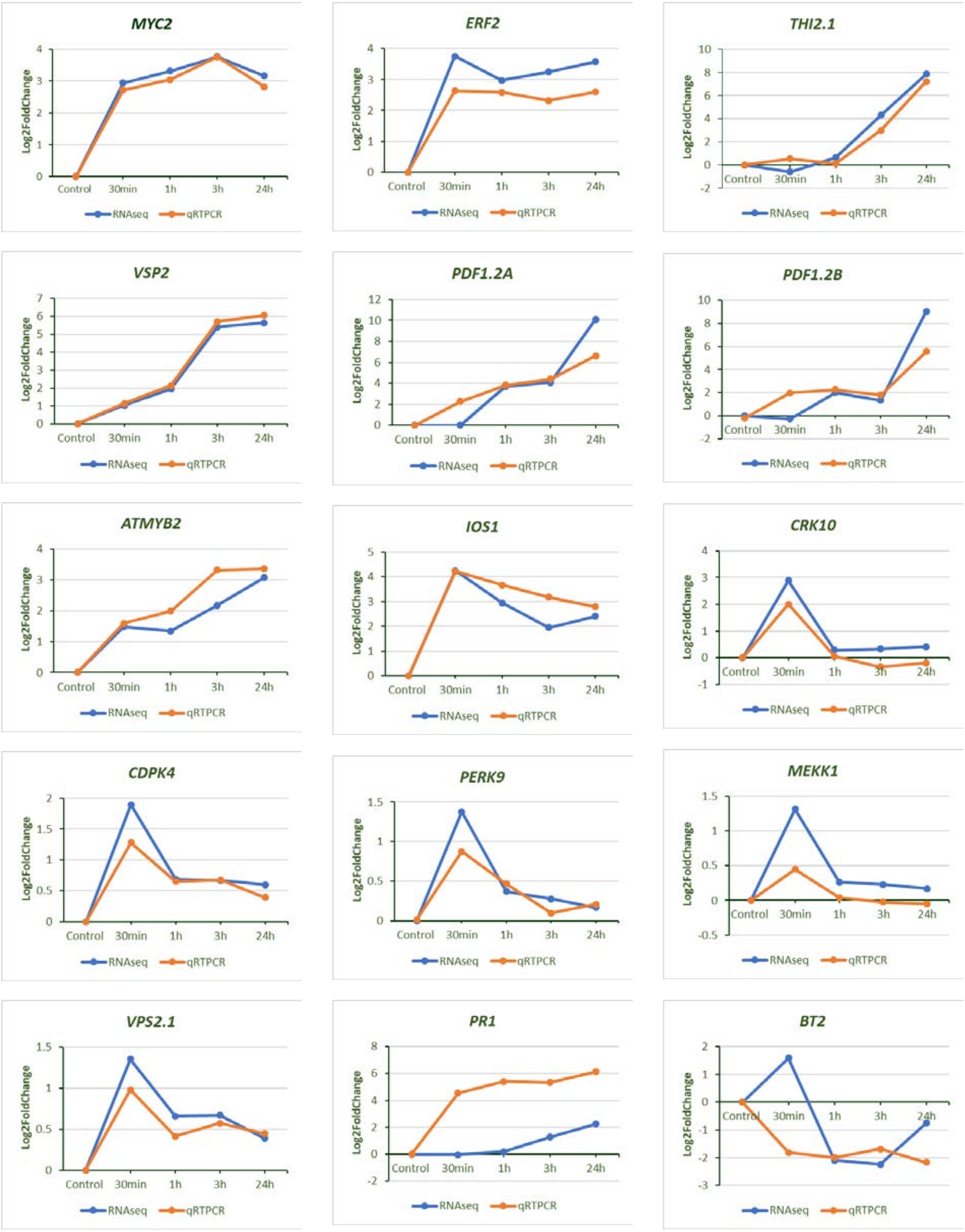

Figure S2. Linear graphics comparing the expression pattern of fifteen selected genes by RNA-seq and RT-qPCR techniques.

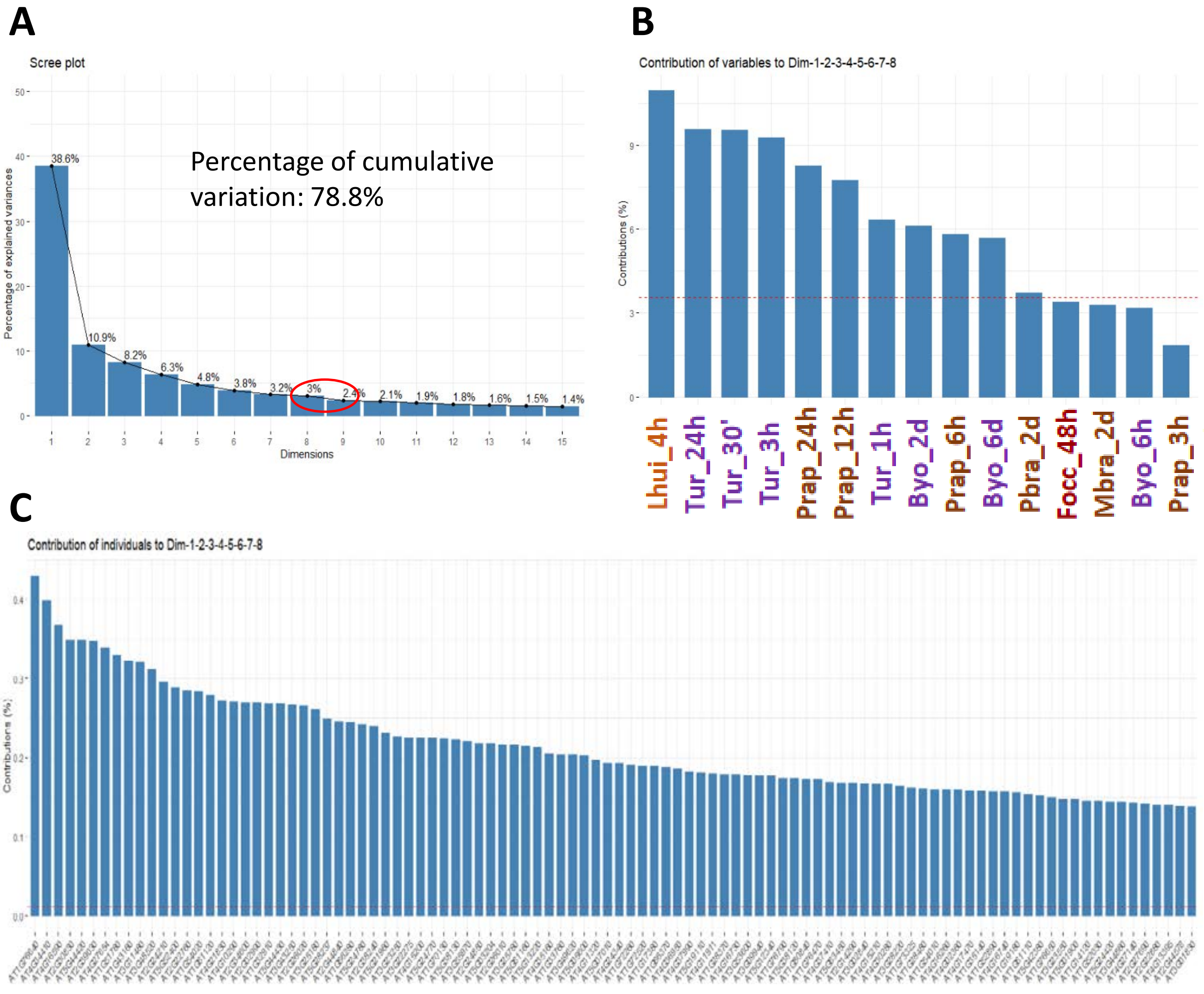

Figure S3. **(A)** Scree plot and percentage of variation explained by each PCA dimension. Red circle indicates the present of a point of inflection. **(B)** Top 15 experiments and **(C)** top 100 genes with the highest contributions in the eight first PCA dimensions. Red dashed lines indicates the mean value of percentage contribution showed through all experiments or genes.

A

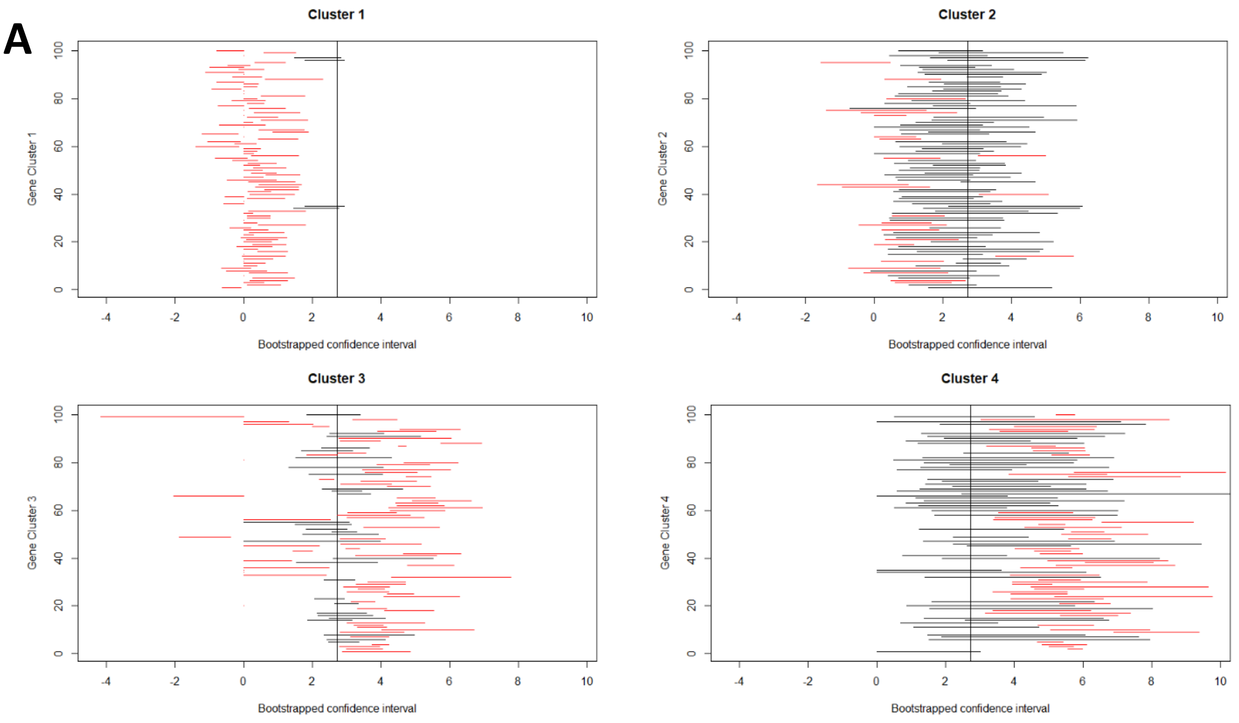

B

Clusters	Number of genes different to the mean
Cluster 1	95 (lower than the mean)
Cluster 2	25 (3 higher than the mean)
Cluster 3	71 (56 higher than the mean)
Cluster 4	49 (higher than the mean)

C

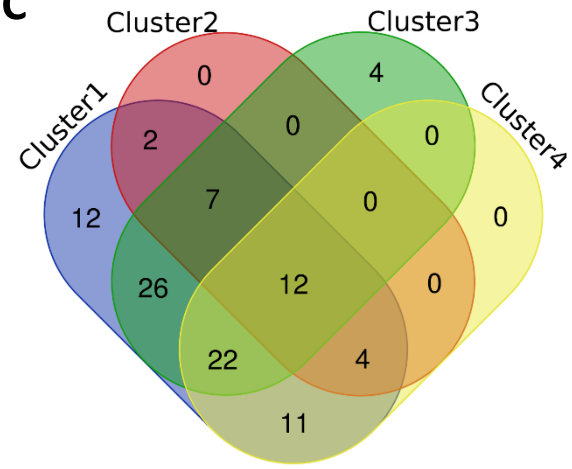

Figure S4. (A) Representation per cluster of the bootstrapped Confidence Intervals against the log2FC mean from the top 100 clustering-responsible genes. Confidence Intervals significantly different from the log2FC mean for each cluster are coloured in red. **(B)** Number of genes significantly different to the log2FC mean in each cluster. **(C)** Venn diagram showing the specific and shared significant genes for each cluster.

Supporting Methods

Real-time RT-qPCR analysis

RT-qPCR assays were performed for validation of the RNA-seq data using a new batch of samples. Rosettes from the ecotype Columbia-0 of *A. thaliana* were sampled and frozen after 0h, 30min, 1h, 3h, and 24h of mite infestation. Total RNA was extracted (Oñate-Sánchez and Vicente-Carbajosa 2008), treated with RQ1 DNase treatment (Promega), and reverse transcribed using Revert Aid^{TM} H Minus First Strand cDNA Synthesis Kit (Thermo Fisher). RT-qPCR was performed for three samples coming from three independent experiments using the Light Cycler 480 SYBR Green I Master (Roche). Specific primers were designed through the PRIMER 3 program (Kõressaar et al. 2018). Primer sequences are indicated in **Table S4**. Gene expression was referred to as fold change ($2^{-\Delta\Delta Ct}$), normalizing the expression to ubiquitin (Livak and Schmittgen 2001). The resulting values were log2 transformed (log2 fold change) for comparison with RNA-seq data. The non-parametric Spearman rank correlation test was used. The statistical analysis was performed using GraphPadPrism v6.01.

Analyses of stored microarray and RNA-seq data

Data analysis was developed according to the different nature of the data. For microarray experiments, raw data was obtained as GenePix (GPR) files for two-channel microarray experiments and chip electronic files (CEL) for single-channel microarrays. Bioconductor, affy and limma packages of R v.3.5.2 were used for background correction, normalization and differential expression analyses (Gautier et al. 2004; Gentleman et al. 2004; Ritchie et al. 2015; R Core Team 2018). A gene expression matrix was generated from affy package, using RMA (robust multiarray average), in the case of the single-channel microarrays (Irizarry et al. 2003). For two-channel microarrays, background correction was performed using the 'normexp' approach (Ritchie et al. 2007) and datasets were log-transformed and normalized using the printtip-loess approach (Smyth and Speed 2003). Then, in both cases, statistical analysis and differential expression were performed using linear models and eBayes functions (Phipson et al. 2016). Adjusted p-values were calculated using Benjamini-Hochberg (BH) FDR correction (Benjamini and Hochberg 1995). DEGs were considered those with a log2 ratio (fold change) higher than 1 and an adjusted p-value < 0.05 for all species, except for *L. huidobrensis*. In this species, to avoid the putative noise inherent to an extremely high number of DEG, we considered a log2 ratio ≥ 2 and an adjusted p-value < 0.01.

For the RNA-seq experiments, raw data were obtained as FASTQ files from the NCBI SRA database (Barrett et al. 2012). Reads were mapped against the TAIR10 reference genome of *A. thaliana* using STAR aligner (v.2.5.3a and v.2.7.0f) (Dobin et al. 2013). The alignments were sorted with Samtools (Li et al. 2009). HTSeq Phyton library was used for the generation of the

count data files from sorted BAM files through the htseq-count script (Anders et al. 2015). RNA-seq read counts were modeled by a generalized linear model considering a specific experimental design for each RNA-seq. To evaluate the DEGs between two different conditions, we performed an analysis with the Bioconductor R package DESeq2 (Love et al. 2014). DEGs were considered those genes with a log2 ratio ≥ 1 and an adjusted p-value < 0.05. These genes were selected for the subsequent comparisons and functional analyses.

Supporting References

Anders S, Pyl PT, Huber W (2015) HTSeq--a Python framework to work with high-throughput sequencing data. **Bioinformatics** 31: 166-169

Barrett T, Clark K, Gevorgyan R, Gorelenkov V, Gribov E, Karsch-Mizrachi I, Kimelman M, Pruitt KD, Resenchuk S, Tatusova T, Yaschenko E, Ostell J (2012) BioProject and BioSample databases at NCBI: facilitating capture and organization of metadata. **Nucleic Acids Res** 40: D57-63

Benjamini Y, Hochberg Y (1995) Controlling the False Discovery Rate: A Practical and Powerful Approach to Multiple Testing. **Journal of the Royal Statistical Society. Series B (Methodological)** 57: 289-300

Dobin A, Davis CA, Schlesinger F, Drenkow J, Zaleski C, Jha S, Batut P, Chaisson M, Gingeras TR (2013) STAR: ultrafast universal RNA-seq aligner. **Bioinformatics** 29: 15-21

Gautier L, Cope L, Bolstad BM, Irizarry RA (2004) affy--analysis of Affymetrix GeneChip data at the probe level. **Bioinformatics** 20: 307-315

Gentleman RC, Carey VJ, Bates DM, Bolstad B, Dettling M, Dudoit S, Ellis B, Gautier L, Ge Y, Gentry J, Hornik K, Hothorn T, Huber W, Iacus S, Irizarry R, Leisch F, Li C, Maechler M, Rossini AJ, Sawitzki G, Smith C, Smyth G, Tierney L, Yang JY, Zhang J (2004) Bioconductor: open software development for computational biology and bioinformatics. **Genome Biol** 5: R80

Irizarry RA, Hobbs B, Collin F, Beazer-Barclay YD, Antonellis KJ, Scherf U, Speed TP (2003) Exploration, normalization, and summaries of high density oligonucleotide array probe level data. **Biostatistics** 4: 249-264

Kõressaar T, Lepamets M, Kaplinski L, Raime K, Andreson R, Remm M (2018) Primer3_masker: integrating masking of template sequence with primer design software. **Bioinformatics** 34: 1937-1938

Li H, Handsaker B, Wysoker A, Fennell T, Ruan J, Homer N, Marth G, Abecasis G, Durbin R, Subgroup GPDP (2009) The Sequence Alignment/Map format and SAMtools. **Bioinformatics** 25: 2078-2079

Livak KJ, Schmittgen TD (2001) Analysis of relative gene expression data using real-time quantitative PCR and the 2(-Delta Delta C(T)) Method. **Methods** 25: 402-408

Love MI, Huber W, Anders S (2014) Moderated estimation of fold change and dispersion for RNA-seq data with DESeq2. **Genome Biol** 15: 550

Oñate-Sánchez L, Vicente-Carbajosa J (2008) DNA-free RNA isolation protocols for Arabidopsis thaliana, including seeds and siliques. **BMC Res Notes** 1: 93

Phipson B, Lee S, Majewski IJ, Alexander WS, Smyth GK (2016) Robust hyperparameter estimation protects against hypervariable genes and improves power to detect differential expression. **Ann Appl Stat** 10: 946-963

R Core Team (2018) R: A language and environment for statistical computing. R Foundation for Statistical Computing, Vienna, Austria.

Ritchie ME, Phipson B, Wu D, Hu Y, Law CW, Shi W, Smyth GK (2015) limma powers differential expression analyses for RNA-sequencing and microarray studies. **Nucleic Acids Res** 43: e47

Ritchie ME, Silver J, Oshlack A, Holmes M, Diyagama D, Holloway A, Smyth GK (2007) A comparison of background correction methods for two-colour microarrays. **Bioinformatics** 23: 2700-2707

Smyth GK, Speed T (2003) Normalization of cDNA microarray data. **Methods** 31: 265-273

Table S1. Enriched biological processes upon 30min, 1h, 3h, and 24h of *T. urticae* infestation.

30min

GOID	GOTerm	Term PValue Corrected	% Associated Genes	Nr. Genes	Associated Genes Found
**GO:0009753**	response to jasmonic acid	5.53E-33	16.74	37	[5PTASE11, ACS6, AGB1, BT2, BT4, ERF1, GL3, GRX480, JAZ1, JAZ10, JAZ12, JAZ3, JAZ5, JAZ6, JAZ7, JAZ8, MPK4, MPK6, MYB3, MYBR1, MYC2, NINJA, NPR1, NPR3, NPR4, PAP1, PEPR1, PFT1, PHYB, RGA1, RGL1, RGL3, SYP121, SYP122, TIFY10B, TIFY7, TPL]
**GO:0009966**	regulation of signal transduction	2.34E-28	10.99	40	[ABI1, AT4G16660, EBF2, EXO70B1, GID1B, HAI1, HY5, JAZ1, JAZ10, JAZ12, JAZ3, JAZ5, JAZ6, JAZ7, JAZ8, KEG, MEKK1, MKK2, MKK4, MKK5, MKK6, MKK9, MKP2, MYB73, MYB77, MYB96, MYBR1, NPR1, NPR3, NPR4, PIL6, PP2CA, PTP1, RCAR3, RGA1, RGL1, RGL3, TIFY10B, TIFY7, TMAC2]
**GO:0009738**	abscisic acid-activated signaling pathway	4.42E-21	12.39	28	[ABF2, ABF3, ABF4, ABI1, BT2, CBL1, CDPK1, CPK32, CPK6, EXO70B1, HAI1, HFR1, HY5, KEG, LTI65, MKK5, MPK3, MPK6, MYB96, NF-YB6, PP2C5, PP2CA, PRN, PYL13, PYL5, PYL7, RCAR3, TMAC2]
**GO:0043549**	regulation of kinase activity	3.42E-14	12.34	19	[AT2G32710, CKS1, CYCA3;1, CYCD5;1, CYCD6;1, CYCP4;1, HB1, ICK1, ICK5, KRP2, MEKK1, MKK2, MKK4, MKK5, MKK6, MKK9, MKP2, PDK1, PTP1]
**GO:0009751**	response to salicylic acid	2.92E-13	10.98	19	[AHBP-1B, BAP1, BAP2, BT1, BT2, BT4, BT5, GRX480, MPK4, MYBR1, NPR1, NPR4, OPR1, RGA1, RGL1, RGL3, SYP121, SYP122, WAK1]
**GO:0023014**	signal transduction by protein phosphorylation	5.92E-12	13.89	15	[MEKK1, MKK2, MKK4, MKK5, MKK6, MKK9, MKP2, MPK11, MPK17, MPK3, MPK4, MPK5, MPK6, PHYB, PTP1]
**GO:0006887**	exocytosis	6.73E-10	17.46	11	[EXO70B1, EXO70B2, EXO70E2, EXO70H4, EXO70H7, RAB8, SEC15B, SEC1B, SEC8, SYP121, SYP122]
**GO:0071482**	cellular response to light stimulus	9.74E-10	10.85	14	[CO, HFR1, HY5, NPH4, PAPP2C, PFT1, PHOT1, PHYB, PIF3, PIL5, PIL6, PKS1, PRN, SIB1]
**GO:0043405**	regulation of MAP kinase activity	4.65E-09	28.57	8	[MEKK1, MKK2, MKK4, MKK5, MKK6, MKK9, MKP2, PTP1]
**GO:0004693**	cyclin-dependent protein serine/threonine kinase activity	2.01E-08	12.64	11	[AT2G32710, CDC2, CKS1, CYCA3;1, CYCD5;1, CYCD6;1, CYCP4;1, ICK1, ICK5, KRP2, ORG1]
**GO:0060548**	negative regulation of cell death	6.20E-08	20.51	8	[AT4G16660, BIR1, BON1, BON2, MKP2, RIN4, SYP121, SYP122]

**GO:0015036**	disulfide oxidoreductase activity	4.97E-07	10.31	10	[AT2G30540, AT4G15660, AT4G15670, AT4G15680, AT4G15700, GRX480, MYB102, NTRC, TH9, TRX5]
**GO:0009740**	gibberellic acid mediated signaling pathway	2.71E-06	12.12	8	[GID1B, HY5, MYB3, PIF3, PIL5, RGA1, RGL1, RGL3]
**GO:0015749**	monosaccharide transmembrane transport	4.50E-06	14.00	7	[AT1G08890, ERD6, ESL1, GLT1, MSS1, PMT5, PMT6]
**GO:0034620**	cellular response to unfolded protein	6.75E-06	12.96	7	[AGB1, BIP3, BZIP28, BZIP60, ERD2, HSP70T-2, mtHsc70-1]
**GO:0010182**	sugar mediated signaling pathway	7.74E-05	11.11	5	[ABF2, ABI5, BT2, EIN3, PAP1]

1h

GOID	GOTerm	Term PValue Corrected	% Associated Genes	Nr. Genes	Associated Genes Found
**GO:0009753**	response to jasmonic acid	5.03E-22	11.31	25	[BT2, BT4, ERF1, GL3, GRX480, JAZ1, JAZ10, JAZ12, JAZ3, JAZ5, JAZ6, JAZ7, JAZ8, MPK6, MYB21, MYBR1, MYC2, NINJA, NPR4, PAP1, PFT1, PHYB, TIFY10B, TIFY7, TPL]
**GO:0071456**	cellular response to hypoxia	3.39E-14	7.63	20	[AGC2-1, AT-HSFB2B, AT1G54050, AT1G59860, AT4G16660, CML37, CML38, HB1, HSFA2, HSP101, HSP17.4, HSP17.6A, HSP17.6II, HSP70, MKK9, PAP1, PBP1, RHL41, STZ, TCH3]
**GO:0009966**	regulation of signal transduction	1.59E-11	5.49	20	[AT4G16660, CPSF30, HSP70, HY5, JAZ1, JAZ10, JAZ12, JAZ3, JAZ5, JAZ6, JAZ7, JAZ8, MKK4, MKK9, MYB73, MYBR1, NPR4, PTP1, TIFY10B, TIFY7]
**GO:0010017**	red or far-red light signaling pathway	8.00E-09	14.29	9	[ELF4, HFR1, HY5, HYH, PAP3, PFT1, PHYB, PIF3, PIL5]
**GO:0031539**	positive regulation of anthocyanin metabolic process	5.81E-08	50.00	5	[GL3, HY5, PAP1, PAP3, PIF3]
**GO:0015036**	disulfide oxidoreductase activity	3.52E-07	9.28	9	[AT1G06830, AT3G62950, AT4G15660, GRX480, GRXS13, GRXS17, NTRC, ROXY2, TRX5]
**GO:0034620**	cellular response to unfolded protein	1.15E-06	12.96	7	[AT3G09440, BIP3, ERD2, HSFA2, HSP70, Hsp70-2, MTHSC70-2]
**GO:0004707**	MAP kinase activity	2.18E-05	11.11	6	[MKK4, MKK9, MPK11, MPK3, MPK6, PTP1]

**GO:0009751**	response to salicylic acid	3.38E-05	5.20	9	[AHBP-1B, BT1, BT2, BT4, CPSF30, GRX480, MYBR1, NPR4, WRKY62]
**GO:0023014**	signal transduction by protein phosphorylation	7.59E-05	6.48	7	[MKK4, MKK9, MPK11, MPK3, MPK6, PHYB, PTP1]
**GO:0043549**	regulation of kinase activity	7.79E-05	5.19	8	[AKINBETA1, CYCA2;3, CYCA3;1, HB1, MKK4, MKK9, PTP1, cycp3;1]
**GO:0009739**	response to gibberellin	1.40E-04	5.65	7	[BT4, GAMMA-TIP, HY5, MYB21, PAP3, PIF3, PIL5]
**GO:0015144**	carbohydrate transmembrane transporter activity	5.68E-04	5.10	5	[AT1G08890, AT1G08900, ESL1, GLT1, STP14]
**GO:0004693**	cyclin-dependent protein serine/threonine kinase activity	6.56E-04	5.75	5	[CDC2, CYCA2;3, CYCA3;1, ORG1, cycp3;1]
**GO:0006475**	internal protein amino acid acetylation	7.05E-04	6.17	5	[BT1, BT2, BT4, HAF01, HD1]

3h

GOID	GOTerm	Term PValue Corrected	% Associated Genes	Nr. Genes	Associated Genes Found
**GO:0009753**	response to jasmonic acid	5.94E-26	12.22	27	[BT2, BT4, ERF1, GL3, GRX480, JAZ1, JAZ10, JAZ12, JAZ3, JAZ5, JAZ6, JAZ7, JAZ8, MPK4, MPK6, MYB21, MYBR1, MYC2, NINJA, PAP1, PEPR1, PFT1, TIFY10B, TIFY7, TPL, TT4, TT8]
**GO:0071456**	cellular response to hypoxia	1.41E-10	6.11	16	[AGC2-1, AT4G16660, CML37, CML38, HB2, HSP101, HSP17.4, HSP17.6A, HSP17.6II, HSP70, PAP1, PBP1, RHL41, S6K2, STZ, TCH3]
**GO:0009962**	regulation of flavonoid biosynthetic process	8.35E-07	17.65	6	[GL3, MYB7, MYC2, PAP1, TT4, TT8]
**GO:0009718**	anthocyanin-containing compound biosynthetic process	1.82E-06	15.38	6	[DFR, GL3, MYC4, PAP1, TT4, TT8]
**GO:0015036**	disulfide oxidoreductase activity	1.97E-06	8.25	8	[AT1G06830, AT1G77370, AT2G30540, AT4G15660, ERO1, GRX480, NTRC, TRX5]
**GO:0009751**	response to salicylic acid	1.38E-05	5.20	9	[BT1, BT2, BT4, BT5, GRX480, KTI1, MPK4, MYBR1, WRKY62]
**GO:0004693**	cyclin-dependent protein serine/threonine kinase activity	1.28E-04	6.90	6	[CDC2, CKS2, CYCA2;3, CYCA3;1, CYCP4;1, ORG1]

**GO:0016573**	histone acetylation	7.94E-04	6.17	5	[BT1, BT2, BT4, BT5, HD1]
**GO:0006626**	protein targeting to mitochondrion	1.33E-03	6.35	4	[AR192, MPPalpha, MYB90, TIM17-1]
**GO:0034620**	cellular response to unfolded protein	1.48E-03	7.41	4	[BIP3, CNX1, HSP70, HSP70T-2]
**GO:0004707**	MAP kinase activity	1.48E-03	7.41	4	[MPK11, MPK3, MPK4, MPK6]

24h

GOID	GOTerm	Term PValue Corrected	% Associated Genes	Nr. Genes	Associated Genes Found
**GO:0009867**	jasmonic acid mediated signaling pathway	6.20E-27	20.00	23	[ERF1, GL3, GRX480, JAZ1, JAZ10, JAZ12, JAZ3, JAZ5, JAZ6, JAZ7, JAZ8, MPK6, MYB21, MYBR1, MYC2, NINJA, PAP1, PFT1, SGT1B, TIFY10B, TIFY7, TPL, TT8]
**GO:0009753**	response to jasmonic acid	7.04E-23	11.31	25	[BT2, BT4, ERF1, GL3, GRX480, JAZ1, JAZ10, JAZ12, JAZ3, JAZ5, JAZ6, JAZ7, JAZ8, MPK6, MYB21, MYBR1, MYC2, NINJA, PAP1, PFT1, SGT1B, TIFY10B, TIFY7, TPL, TT8]
**GO:0015036**	disulfide oxidoreductase activity	5.24E-10	11.34	11	[AT2G30540, AT2G47870, AT3G21460, AT4G15660, GRX480, GRXC2, NTRC, ROXY2, TH8, TRX3, TRX5]
**GO:0009751**	response to salicylic acid	1.87E-08	6.94	12	[BT1, BT2, BT4, BT5, GRX480, KTI1, MKK7, MYBR1, WRKY18, WRKY38, WRKY40, WRKY62]
**GO:0015144**	carbohydrate transmembrane transporter activity	1.72E-07	9.18	9	[AT1G08890, AT5G61520, ESL1, GLT1, INT2, MSS1, PMT5, PMT6, SFP2]
**GO:0071456**	cellular response to hypoxia	1.48E-06	4.58	12	[AGC2-1, CML37, CML38, HSP101, HSP70, MKK9, PAP1, PBP1, RHL41, S6K2, STZ, TCH3]
**GO:0004707**	MAP kinase activity	1.39E-05	11.11	6	[MKK7, MKK9, MPK11, MPK3, MPK6, PTP1]
**GO:0009738**	abscisic acid-activated signaling pathway	2.01E-05	4.42	10	[BT2, CEN2, HAI1, HFR1, KEG, MPK3, MPK6, NF-YB6, PP2CA, PRN]
**GO:0009962**	regulation of flavonoid biosynthetic process	2.26E-05	14.71	5	[GL3, MYB7, MYC2, PAP1, TT8]
**GO:0023014**	signal transduction by protein phosphorylation	4.57E-05	6.48	7	[MKK7, MKK9, MPK11, MPK3, MPK6, PHYA, PTP1]

**GO:0016573**	histone acetylation	8.85E-05	7.41	6	[BT1, BT2, BT4, BT5, HAF01, HD1]
**GO:0009863**	salicylic acid mediated signaling pathway	3.82E-04	7.25	5	[GRX480, MKK7, MYBR1, WRKY38, WRKY62]
**GO:0010017**	red or far-red light signaling pathway	2.05E-03	6.35	4	[ELF4, HFR1, PFT1, PHYA]
**GO:0009734**	auxin-activated signaling pathway	2.67E-03	4.40	4	[BT2, MYB73, MYBR1, SGT1B]
**GO:1902531**	regulation of intracellular signal transduction	2.90E-03	5.19	4	[HSP70, MKK7, MKK9, PTP1]

Table S2. Selected transcriptomic experiments for the analysis of the response of plants to herbivore attack. Accession number of the experiment is provided as Gene Expression Datasets Series (GSE) from the Gene Expression Omnibus (GEO) platform, as E-MTAB accession number from ArrayExpress database, as SRP accession number from the Sequence Read Archive (SRA) or as NASCarray experiments from the Nottingham Arabidopsis Stock Centre's microarray database. The presence of an asterisk (*) indicates that a re-analysis of the data was performed.

Accession	Year	Herbivore	Type of herbivore	Type of experiment	Infestation time	Plant tissue	Plant age	Ref.
GSE38281*	2014	*Liriomyza huidobrensis*	Leafminer (Dipteran)	Microarray	4h	Leaves	4 weeks	(Zhang et al. 2012)
GSE39245*	2013	*Brevicoryne brassicae*	Aphid	Microarray	72h	Rosettes	4.5 weeks	(Barah et al. 2013)
E-MTAB-3223	2015	*Myzus persicae*<br>*Myzus cerasi*<br>*Rhopalosiphum pisum*	Aphids	Microarray	3, 6, 24h	Rosettes	4 weeks	(Jaouannet et al. 2015)
NASC-330*	2005	*Frankiniella occidentalis*	Thrip	Microarray	24, 48h	Leaves	5 weeks	(De Vos et al. 2005)
GSE6516	2006	*Bemisia tabaci*	Hemiptera	Microarray	21d	Leaves	4 weeks	(Kempema et al. 2007)
GSE114211	2019	*Pieris brassicae*<br>*Mamestra brassicae*	Lepidopterans	Microarray	48h	Leaves	8 weeks	(Oberländer et al. 2019)
E-MTAB-1418	2013	*Spodoptera littoralis*	Lepidopteran	Microarray	8d	Leaves	4 weeks	(Schweizer et al. 2013)
SRP073212	2016	*Pieris rapae*	Lepidopteran	RNA-seq	3, 6, 12, 24h	Leaves	5 weeks	(Coolen et al. 2016)
SRP144249	2018	*Brevipalpus yothersi*	Mite	RNA-seq	6h, 2d, 6d	Leaves	4 weeks	(Arena et al. 2018)
-	2020	*Tetranychus urticae*	Mite	RNA-seq	30 min<br>1, 3, 24h	Rosettes	4 weeks	-

Supporting References:

Arena GD, Ramos-González PL, Rogerio LA, Ribeiro-Alves M, Casteel CL, Freitas-Astúa J, Machado MA (2018) Making a Better Home: Modulation of Plant Defensive Response by Brevipalpus mites. **Front Plant Sci** 9: 1147

Barah P, Winge P, Kusnierczyk A, Tran DH, Bones AM (2013) Molecular signatures in Arabidopsis thaliana in response to insect attack and bacterial infection. **PLoS One** 8: e58987

Coolen S, Proietti S, Hickman R, Davila Olivas NH, Huang PP, Van Verk MC, Van Pelt JA, Wittenberg AH, De Vos M, Prins M, Van Loon JJ, Aarts MG, Dicke M, Pieterse CM, Van Wees SC (2016) Transcriptome dynamics of Arabidopsis during sequential biotic and abiotic stresses. **Plant J** 86: 249-267

De Vos M, Van Oosten VR, Van Poecke RM, Van Pelt JA, Pozo MJ, Mueller MJ, Buchala AJ, Métraux JP, Van Loon LC, Dicke M, Pieterse CM (2005) Signal signature and transcriptome changes of Arabidopsis during pathogen and insect attack. **Mol Plant Microbe Interact** 18: 923-937

Jaouannet M, Morris JA, Hedley PE, Bos JI (2015) Characterization of Arabidopsis Transcriptional Responses to Different Aphid Species Reveals Genes that Contribute to Host Susceptibility and Non-host Resistance. **PLoS Pathog** 11: e1004918

Kempema LA, Cui X, Holzer FM, Walling LL (2007) Arabidopsis transcriptome changes in response to phloem-feeding silverleaf whitefly nymphs. Similarities and distinctions in responses to aphids. **Plant Physiol** 143: 849-865

Oberländer J, Lortzing V, Hilker M, Kunze R (2019) The differential response of cold-experienced Arabidopsis thaliana to larval herbivory benefits an insect generalist, but not a specialist. **BMC Plant Biol** 19: 338

Schweizer F, Fernández-Calvo P, Zander M, Diez-Diaz M, Fonseca S, Glauser G, Lewsey MG, Ecker JR, Solano R, Reymond P (2013) Arabidopsis basic helix-loop-helix transcription factors MYC2, MYC3, and MYC4 regulate glucosinolate biosynthesis, insect performance, and feeding behavior. **Plant Cell** 25: 3117-3132

Zhang S, Zhang Z, Kang L (2012) Transcriptome response analysis of Arabidopsis thaliana to leafminer (Liriomyza huidobrensis). **BMC Plant Biol** 12: 234

Table S3. Individual genes significantly deregulated in clusters 3 and 4.

Gene ID	Cluster 1	Cluster 2	Cluster 3	Cluster 4	Gene name	Description
AT5G42380	-1.99	-3.28	-0.43	2.72	CML37	Calcium-binding protein CML37 (Q9FIH9)
AT3G01830	-1.89	-2.38	-0.94	2.38	CML40	Probable calcium-binding protein CML40 (Q9SGI8)
AT1G72520	-2.36	-1.11	#N/A	3.24	LOX4	Lipoxygenase 4, chloroplastic (Q9FNX8)
AT3G48520	-2.81	-1.75	#N/A	5.67	CYP94B3	CYP94B3 (A0A178V8H3)
AT4G30280	-2.73	-2.21	#N/A	2.70	XTH18	Xyloglucan endotransglucosylase/hydrolase 18 (Q9M0D2)
AT2G22880	-2.38	-1.46	#N/A	2.47	-	At2g22880 (O81005)
AT2G38240	-2.82	#N/A	-2.04	4.08	ANS	2-oxoglutarate-dependent dioxygenase ANS (O80449)
AT5G05600	-2.73	#N/A	-0.36	2.95	-	2-oxoglutarate-dependent dioxygenase At5g05600 (Q9FFF6)
AT2G27690	-2.81	#N/A	-1.08	2.05	CYP94C1	Cytochrome P450 94C1 (Q9ZUX1)
AT2G24850	-2.04	#N/A	-1.18	2.67	TAT3	Probable aminotransferase TAT3 (Q9SK47)
AT3G44860	-2.64	#N/A	#N/A	2.38	FAMT	Farnesoic acid carboxyl-O-methyltransferase (Q9FYC4)
AT5G13220	-2.59	#N/A	#N/A	2.40	JAZ10	jasmonate-zim-domain protein 10 (cc:AT5G13220)
AT5G63450	-2.86	#N/A	#N/A	2.77	CYP94B1	cytochrome P450, fam 94, subfam B, polypep 1 (AT5G63450)
AT3G25780	-2.06	#N/A	#N/A	3.47	AOC3	Allene oxide cyclase 3, chloroplastic (Q9LS01)
AT5G12340	-2.60	#N/A	#N/A	2.66	-	DUF4228 domain protein (Q1PDX4)
AT1G76470	-2.63	#N/A	#N/A	2.66	-	NAD-binding Rossmann-fold superfamily protein (F4I2E5)
AT2G34600	-2.29	#N/A	#N/A	4.52	TIFY 5B	Protein TIFY 5B (O64687)
AT3G25180	-2.52	#N/A	#N/A	4.34	CYP82G1	Cytochrome P450 82G1 (Q9LSF8)
AT1G61120	-1.89	#N/A	#N/A	3.55	GES	(E,E)-geranyllinalool synthase (Q93YV0)
AT1G28480	-1.81	#N/A	#N/A	2.33	GRXC9	Glutaredoxin-C9 (Q9SGP6)
AT1G66280	-2.60	#N/A	#N/A	2.83	BGLU22	Beta-glucosidase 22 (Q9C8Y9)

Gene ID	Cluster 1	Cluster 2	Cluster 3	Cluster 4	Gene name	Description
AT5G21960	-1.30	-1.63	3.68	#N/A	ERF016	Ethylene-responsive transcription factor ERF016 (Q9C591)
AT4G37990	-2.60	-1.41	2.98	#N/A	CAD8	Cinnamyl alcohol dehydrogenase 8 (Q02972)
AT4G15200	-2.73	-2.30	3.20	#N/A	AFH3	formin 3 (cc:AT4G15200)
AT1G33760	-2.10	-2.01	1.11	#N/A	ERF022	Ethylene-responsive transcription factor ERF022 (Q9LQ28)
AT1G70130	-2.04	-2.34	1.01	#N/A	LECRK52	L-type lectin-domain containing receptor kinase V.2 (O04533)
AT4G13395	-1.78	-2.07	2.36	#N/A	DVL10	DVL10 (Q6IM91)
AT3G11480	-2.36	#N/A	1.52	#N/A	BSMT1	Salicylate/benzoate carboxyl methyltransferase (Q6XMI3)
AT4G11320	-2.69	#N/A	3.11	#N/A	RDL5	Probable cysteine protease RDL5 (Q9SUS9)
AT1G52030	-2.52	#N/A	1.11	#N/A	F-ATMBP	Myrosinase-binding protein 2 (Q9SAV1)
AT4G21830	-1.61	#N/A	1.02	#N/A	MSRB7	Peptide methionine sulfoxide reductase B7 (Q8VY86)
AT1G54010	-2.45	#N/A	1.76	#N/A	GLL23	Inactive GDSL esterase/lipase-like protein 23 (Q8W4H8)
AT5G24770	-2.73	#N/A	2.04	#N/A	VSP2	Vegetative storage protein 2 (O82122)
AT4G24340	-2.30	#N/A	1.74	#N/A	-	At4g24340 (Q8GW53)
AT1G73325	-2.13	#N/A	0.74	#N/A	-	Kunitz family trypsin (Q9FX28)
AT5G24420	-2.66	#N/A	0.76	#N/A	PGL5	Probable 6-phosphogluconolactonase 3 (Q8LEV7)
AT4G17470	-2.64	#N/A	2.11	#N/A	-	Alpha/beta-Hydrolases superfamily protein (Q501G7)
AT2G24210	-2.64	#N/A	3.86	#N/A	TPS10	Tricyclene synthase, chloroplastic (Q9ZUH4)
AT4G35160	-2.83	#N/A	2.09	#N/A	-	O-methyltransferase family protein (Q9T003)
AT4G37410	-2.71	#N/A	2.15	#N/A	CYP81F4	Cytochrome P450 81F4 (Q9SZU1)
AT5G07010	-3.10	#N/A	0.46	#N/A	SOT15	Sulfotransferase (A0A178UG65)
AT4G11911	-3.16	#N/A	2.43	#N/A	-	unknown protein (AT4G11911)
AT2G44578	-2.30	#N/A	1.92	#N/A	-	RING/U-box domain-containing protein (A8MS73)
AT4G10290	-3.29	#N/A	2.37	#N/A	-	RmlC-like cupins superfamily protein (F4JLM2)
AT1G14120	-2.92	#N/A	1.42	#N/A	-	2-oxoglutarate and Fe(II)-dependent oxygenase (Q9XI76)
AT3G55970	-2.60	#N/A	1.19	#N/A	JRG21	JRG21 (A0A178V764)
AT4G15210	-3.25	#N/A	2.42	#N/A	BAM5	Beta-amylase 5 (P25853)
AT5G44420	#N/A	#N/A	-1.49	#N/A	PDF1.2A	Defensin-like protein 16 (Q9FI23)
AT5G44430	#N/A	#N/A	-2.08	#N/A	PDF1.2C	PDF1.2c (A0A178UR42)
AT2G26020	#N/A	#N/A	-2.73	#N/A	PDF1.2B	PDF1.2b (A0A178VQC3)
AT2G26010	#N/A	#N/A	-2.73	#N/A	PDF1.3	PDF1.3 (A0A178VSS6)

Gene ID	Cluster 1	Cluster 2	Cluster 3	Cluster 4	Gene name	Description
AT1G43160	-2.88	-1.96	1.21	5.38	RAP2-6	Ethylene-responsive transcription factor RAP2-6 (Q7G1L2)
AT4G27654	-1.93	-1.88	2.41	4.91	-	At4g27654 (Q3E9V5)
AT1G76650	-2.45	-1.79	1.90	2.12	CML38	CML38 (A0A178WMC5)
AT1G80840	-1.77	-1.89	1.09	2.44	WRKY40	Probable WRKY transcription factor 40 (Q9SAH7)
AT3G02840	-1.71	-3.15	0.50	2.56	-	ARM repeat superfamily protein (Q9M8S5)
AT2G02990	-2.60	-1.89	0.95	4.50	RNS1	Ribonuclease 1 (P42813)
AT1G52890	-2.73	-1.75	1.02	2.63	NAC019	NAC domain-containing protein 19 (Q9C932)
AT1G19180	-2.53	-1.32	0.71	2.61	TIFY10A	TIFY10A (A0A178W7G1)
AT1G28370	-2.15	-1.31	1.24	2.65	ERF11	ERF domain protein 11 (cc:AT1G28370)
AT3G23250	-1.57	#N/A	0.81	3.46	MYB15	Transcription factor MYB15 (Q9LTC4)
AT3G43250	-2.30	#N/A	2.05	3.61	-	Coiled-coil protein (DUF572) (Q9LXK9)
AT5G38120	-3.02	#N/A	2.20	1.83	4CLL8	4-coumarate--CoA ligase-like 8 (Q84P26)
AT5G19110	-2.84	#N/A	1.91	1.53	-	Eukaryotic aspartyl protease family protein (cc:AT5G19110)
AT3G53600	-2.64	#N/A	1.71	2.24	-	At3g53600 (Q9LFG0)
AT1G51780	-2.60	#N/A	2.98	4.12	ILL5	IAA-amino acid hydrolase ILR1-like 5 (Q9SWX9)
AT1G76640	-2.14	#N/A	2.58	5.15	CML39	Calcium-binding protein CML39 (Q9SRE7)
AT2G22760	-2.38	#N/A	1.51	4.10	BHLH19	Putative uncharacterized protein At2g22760 (C0SV56)
AT1G76790	-2.73	#N/A	0.89	1.76	IGMT5	Indole glucosinolate O-methyltransferase 5 (Q9SRD4)
AT1G17380	-2.32	#N/A	0.81	2.94	TIFY11A	Protein TIFY 11A (Q9LDU5)
AT1G66370	-2.52	#N/A	2.23	2.00	MYB113	Transcription factor MYB113 (Q9FNV9)
AT5G52400	-2.73	#N/A	1.09	4.16	CYP715A1	Cytochrome P450, fam 715, subfam A, polypep 1 (F4KG63)
AT2G44840	-1.62	#N/A	2.90	2.72	ERF13	Ethylene-responsive transcription factor 13 (Q8L9K1)
AT2G14290	-2.73	#N/A	1.47	1.65	-	F-box protein At2g14290 (Q9ZQ60)
AT2G30830	-2.73	#N/A	2.74	4.62	-	2-oxoglutarate and Fe(II)-dependent oxygenase (O80850)
AT4G34410	-2.13	#N/A	1.60	5.73	ERF109	Ethylene-responsive transcription factor ERF109 (Q9SZ06)
AT5G38130	-2.76	#N/A	2.82	2.21	-	At5g38130 (Q6DBD5)
AT4G02360	-2.56	#N/A	1.12	1.83	-	Putative uncharacterized protein AT4g02360 (O81297)

Table S4. Oligonucleotide sequences for RT-qPCR analysis. F, forward; R, reverse.

ID	Nombre	Secuencia	
At2g47190	ATMYB2	CCTCAAACTCCATTCTCTTTGG	F
		TCTTAGGTGTTTGGCTTGCTTT	R
At4g09570	CDPK4	TCAAGAAAATGGCCTTACGAGT	F
		TTCAAAGGTGATTGTTCCACTG	R
At2g06530	VPS2.1	AATGAACAGGCAGATGAACCTT	F
		CTTCTTCCTCATCTCCTTCCAA	R
At4g08500	MEKK1	GGAAGAGATGGGAGCTAGGTTT	F
		CCATCTGGATAGATAGGCGAAG	R
At5g47220	ERF2	GGGTTAATTCCGGTGAACCT	F
		CACGTGTCTCATCACCAACC	R
At1g51800	IOS1	GCGCTACCACGAAAAAGAAG	F
		AGGTTCAAAACCGTGGTGAG	R
At4g23180	CRK10	TAACGGGCGACCATTAGAAC	F
		GGCACGGGTAAAGTCACAGT	R
At3g48360	BT2	AAGAAAAGAAGGCGACGACA	F
		AACGCCTTACAAGGCTCTGA	R
At1g68690	PERK9	CTCCGGTTTCAAATTCTCCA	F
		TGGTGGTGGTGAGGTAGTCA	R
At1g32640	MYC2	TCCGAGTCCGGTTCATTCT	F
		TCTCGGGAGAAAGTGTTATTGAA	R
At5g24770	VSP2	ATGCCAAAGGACTTGCCCTA	F
		CGGGTCGGTCTTCTCTGTTC	R
At5g44420	PDF1.2A	GTTCTCTTTGCTGCTTTCGAC	F
		GCAAACCCCTGACCATGT	R
At2g26020	PDF1.2B	GTTCTCTTTGCAGCTTTTG	F
		TCCACCATTGTTGGTACTT	R
At2g14610	PR1	TCAGTGAGACTCGGATGTGC	F
		CGTTCACATAATTCCCACGA	R
At1g72260	THI2.1	CTGCCCTTCCAACCAAGCTA	F
		TGGCATCCACTCACTTGCAT	R
